# The protein tyrosine phosphatase CD45 promotes PMN transepithelial migration, antimicrobial function, and colonic mucosal repair

**DOI:** 10.1172/jci.insight.203438

**Published:** 2026-06-23

**Authors:** Jael Miranda, Dylan J. Fink, Zachary S. Wilson, Roland Hilgarth, Asma Nusrat, Charles A. Parkos, Jennifer C. Brazil

**Affiliations:** Department of Pathology, University of Michigan Medical School, Ann Arbor, Michigan, USA.

**Keywords:** Immunology, Inflammation, Neutrophils

## Abstract

Polymorphonuclear neutrophils (PMNs) serve as frontline defenders against injury and infection, eliminating pathogens and initiating mucosal tissue repair. However, excessive PMN transepithelial migration (TEpM) contributes to chronic mucosal inflammatory disorders, including inflammatory bowel disease. PMN proinflammatory and pro-repair functions are regulated by incompletely defined signaling cascades involving kinases and phosphatases. Here, we determined how the protein tyrosine phosphatase CD45/PTPRC regulates PMN trafficking and effector functions in the gut. Pharmacologic inhibition of CD45 significantly reduced PMN colonic TEpM in vitro and in vivo and decreased intestinal PMN trafficking was observed in transgenic mice with PMN-specific deletion of *Cd45* (*MRP8-Cre;Cd45^fl/fl^*). Beyond limiting TEpM, CD45 depletion impaired key antimicrobial functions, including degranulation and phagocytosis, indicating broader effects on PMN effector activity. Importantly, recovery from dextran sodium sulfate–induced colitis and biopsy-induced colonic wounding was delayed in *MRP8-Cre;Cd45^fl/fl^* mice, linking altered PMN function to defective mucosal healing. Mechanistically, CD45 depletion reduced surface expression of the β2 integrin CD11b/CD18 and inactivated the Src family kinase member Lyn. Together, these data highlight an important CD45/CD11b/Lyn signaling axis that regulates PMN trafficking and effector functions in the intestine and identify CD45 as a promising target for modulating PMN function to promote mucosal tissue repair.

## Abstract 2

CD45 allows neutrophils to move effectively into injured intestinal tissues where they fight infection, and drive critical mucosal repair processes.

## Graphical abstract

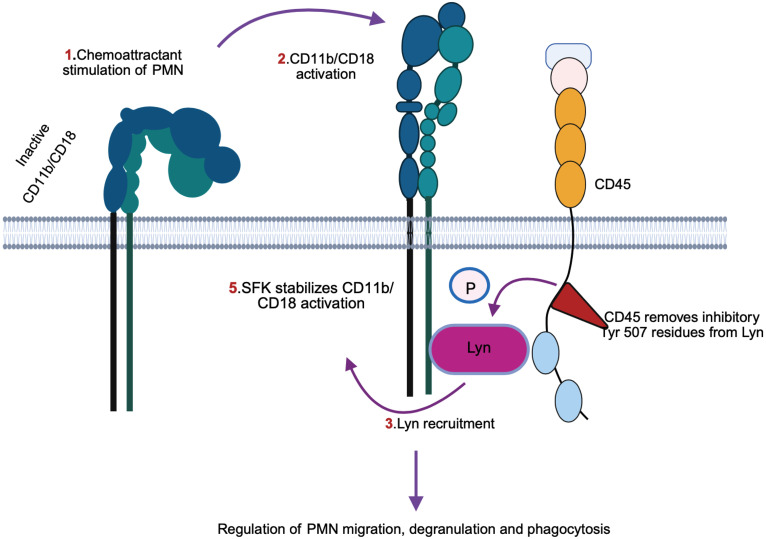

## Introduction

Polymorphonuclear neutrophils (PMNs) are the first immune responders to injury or infection, playing critical roles in clearing invading pathogens and initiating subsequent tissue repair processes (1, 2). Epithelial surfaces of mucosal tissues (including the lungs and intestine) serve as barriers against pathogens and environmental insults, and along with PMNs, play a pivotal role in restoration of mucosal integrity following injury/inflammation (3–5). PMN effector functions are regulated by incompletely understood interactions between surface receptors (including β2 integrins) and intracellular signaling cascades orchestrated by phosphatases and kinases. Specifically, protein tyrosine phosphatases (PTPs), including membrane-bound receptor-like PTPs, dephosphorylate tyrosine residues on intracellular kinases to regulate downstream cell signaling relays (6).

PTP receptor type C (PTPRC), better known as CD45, is expressed by all nucleated hematopoietic cells but has almost exclusively been studied in the context of T and B cell adaptive immune function (7, 8). While its functions in lymphocyte activation are somewhat well characterized, far less is known about the role CD45 plays in mediating PMN responses within inflammatory environments. In this study, we investigated the role of CD45 in orchestrating PMN trafficking and effector functions during mucosal inflammation and subsequent tissue repair in the gut.

We demonstrate that inhibition or depletion of CD45 reduces PMN intestinal trafficking in vitro and in vivo. Furthermore, our results show that inhibition or deletion of CD45 diminishes PMN phagocytosis and degranulation responses. Importantly, mice with PMN-specific knockdown of CD45 exhibited delayed recovery from dextran sodium sulfate–induced (DSS-induced) colitis and from biopsy-based wounding, highlighting that CD45-mediated regulation of PMN recruitment and function is important for intestinal repair. The data also revealed that PMNs deficient in CD45 had reduced surface expression and activation of the β2 integrin CD11b/CD18, a known regulator of PMN trafficking and other effector functions (9–11). Finally, we observed that during PMN activation, CD45 plays a critical role in removing the inhibitory phosphate group from tyrosine 507 (Tyr507) on the Src family kinase (SFK) member Lyn. Taken together, our results reveal a crucial signaling axis whereby CD45 dephosphorylates Lyn kinase to increase PMN CD11b/CD18 surface expression and activation and positively regulate PMN trafficking and antimicrobial functions in mucosal tissues. Overall, we show that CD45 serves as a critical regulator of neutrophil plasticity, influencing not only proinflammatory capabilities but also reparative functions required for resolution of inflammation and effective tissue healing. Our findings offer important insights into mucosal immunology and lay the groundwork for developing targeted therapies that harness CD45-mediated signaling pathways to alleviate chronic inflammation and promote mucosal healing.

## Results

### CD45 regulates PMN intestinal transepithelial migration in vitro and in vivo.

**Figure 1:**
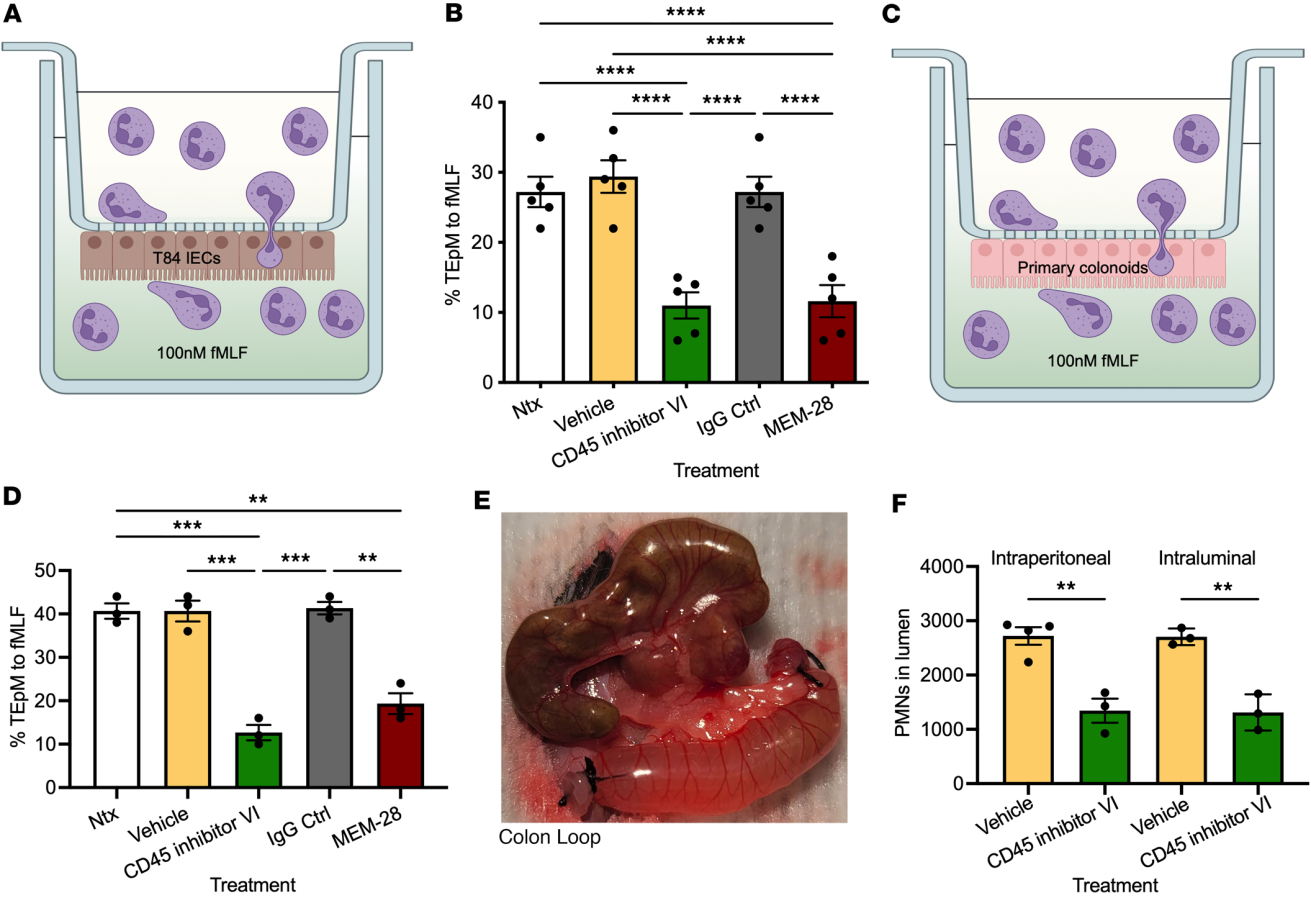
CD45 inhibition blocks PMN TEpM in vitro and in vivo. (**A** and **B**) Human PMNs (1 × 10^6^) were incubated with 500 nM CD45 inhibitor VI or vehicle control or 10 μg/mL anti-CD45 mAb (MEM-28) or isotype-matched control IgG mAb before addition to the basolateral surface of confluent inverted T84 monolayers. PMNs migrated in the physiologically relevant basolateral to apical direction for 1 hour in response to a 100 nM gradient of *N*-formyl-L-methionyl-L-leucyl-L-phenylalanine (fMLF). Numbers of migrated PMNs were quantified by myeloperoxidase assay (*n* = 5 independent experiments). (**C** and **D**) Human PMNs (1 × 10^6^) were incubated with 500 nM CD45 inhibitor VI, vehicle control, 10 μg/mL anti-CD45 mAb MEM-28, or isotype-matched control IgG mAb before addition to the basolateral surface of confluent inverted human colonoid–derived monolayers of primary IECs. PMNs migrated for 1 hour in response to a 100 nM gradient of fMLF (*n* = 3 independent experiments). (**E** and **F**) Quantification of absolute number of PMNs recruited into the lumen of proximal colon loops following intraluminal injection of 1 nM LTB_4_ ± 500 nM CD45 inhibitor VI or intraperitoneal injection of 3 mg/kg body weight CD45 inhibitor VI (*n* = 3–4 independent experiments). Data are shown as mean ± SEM and were analyzed by 1-way ANOVA with Tukey’s post hoc testing. **P < 0.01; ***P < 0.001; ****P < 0.0001.

PMN trafficking is facilitated by a complex series of incompletely understood signaling cascades that are regulated by adhesion molecules, including integrins as well as intracellular phosphatases and kinases. Given the abundant expression of the phosphatase CD45 by PMN (and the lack of knowledge in terms of how it regulates PMN function), studies were performed to determine effects of CD45 phosphatase inhibition on PMN transepithelial migration (TEpM). The intracellular domain of CD45 contains physiologically active phosphatase (D1) and inactive phosphatase (D2) motifs that are linked together in a fixed orientation crucial for CD45 substrate binding and phosphatase function. To determine the importance of CD45 phosphatase activity during PMN TEpM, human PMNs were incubated with an inhibitor of CD45 phosphatase activity (CD45 inhibitor VI) that binds in an irreversible manner to an allosteric pocket at the D1-D2 domain interface of CD45 (12). Dose-response studies revealed that exposure of human PMNs to 100–250 nM CD45 inhibitor VI did not have a significant effect on *N*-formyl-L-methionyl-L-leucyl-L-phenylalanine–driven (fMLF-driven) migration across T84 intestinal epithelial cell (IEC) monolayers in the physiologically relevant basolateral to apical direction (Supplemental Figure 1A). However, at a concentration of 500 nM, CD45 inhibitor VI reduced detectable PMN numbers in the apical chamber by 60% or more compared with vehicle control (Supplemental Figure 1A, *P* < 0.05; Figure 1, A and B, *P* < 0.0001; supplemental material available online with this article; https://doi.org/10.1172/jci.insight.203438DS1). Importantly, previous studies have indicated that incubation with 500 nM CD45 inhibitor VI resulted in no detectable cytotoxicity in T cells cultured in vitro (12). Similar to effects observed with CD45 inhibitor VI, incubation of PMNs with an anti-CD45 mAb (MEM-28; 10 mg/mL), that binds to the extracellular domain of human CD45, reduced PMN TEpM by 50% or more compared with IgG control–treated PMNs (Figure 1, A and B, *P* < 0.0001).

We next assessed effects of CD45 inhibition on PMN chemotaxis across collagen-coated Transwells. As can be seen in Supplemental Figure 1B, incubation of human PMNs with 500 nM CD45 inhibitor VI significantly reduced chemotaxis to fMLF relative to PMNs treated with vehicle control (*P* < 0.01). To determine whether decreased PMN TEpM was due to possible off-target effects of CD45 inhibitor VI on epithelial barrier function, trans-epithelial electrical resistance (TEER) of inverted T84 IEC monolayers was measured before and after exposure to 500 nM CD45 inhibitor VI. As can be seen in Supplemental Figure 1C, exposure to 500 nM CD45 inhibitor VI for 1 hour had no effect on IEC TEER/barrier function, suggesting that the observed CD45-mediated reductions in TEpM are mediated through PMN-intrinsic mechanisms. We next assessed effects of CD45 inhibition on migration of human PMNs across cultured monolayers of primary human colonic epithelial cells (colonoids). Human colonoids grown as inverted monolayers on permeable Transwell supports developed robust barriers, with TEER readings between 900 and 1000 Ω•cm^2^ observed on day 5 after seeding (Supplemental Figure 1D, *P* < 0.0001). While the T84 IEC model of PMN TEpM is well established (9, 13, 14), these data represent the first report to our knowledge of robust migration of human PMNs across primary non-transformed colonic IECs. Importantly, exposure of PMNs to 500 nM CD45 inhibitor VI or 10 mg/mL MEM-28 significantly reduced migration of PMNs across inverted monolayers of primary human colonoid–derived IECs relative to PMNs exposed to vehicle or IgG controls (Figure 1, C and D, *P* < 0.001 and *P* < 0.01). We next extended in vitro migration experiments to in vivo animal studies. For these experiments, a previously established proximal surgical loop model was utilized that enables quantitative and spatiotemporal studies of PMN trafficking across colonic mucosa in response to luminally administered chemoattractants (15, 16). Analysis of PMN migration into the proximal colon in response to luminally applied leukotriene B4 (LTB_4_) revealed that CD45 inhibition resulted in a 50% or greater decrease in the number of PMNs reaching the intestinal lumen, relative to mice injected with vehicle control (Figure 1, E and F, *P* < 0.01). Importantly, a similar decrease in PMN trafficking was observed when CD45 inhibitor VI was administered by intraluminal (500 mM) or intraperitoneal (3 mg/kg body weight) injection. Taken together, these data demonstrate that CD45 phosphatase activity is required for effective PMN TEpM in vivo and in vitro.

### CD45 regulates PMN degranulation and phagocytosis responses.

**Figure 2:**
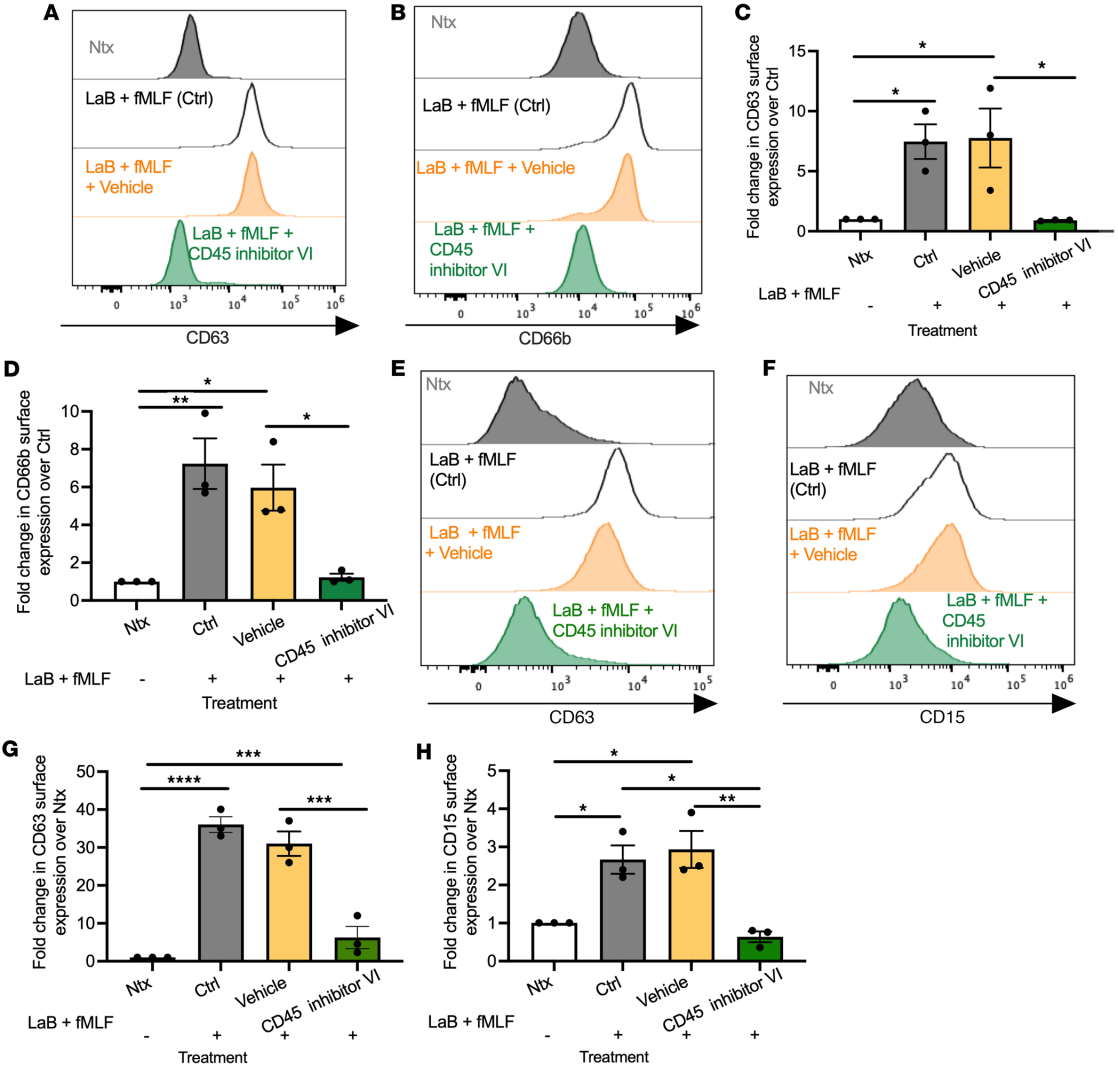
CD45 inhibition decreases degranulation in human and murine PMNs. (**A**–**D**) Human PMNs were exposed to 500 nM CD45 inhibitor VI, vehicle control (Vehicle), or Hanks balanced salt solution with Ca^2+^ and Mg^2+^ (HBSS^+^) HBSS^+^ (Ctrl) for 30 minutes at 37°C followed by stimulation with 1.25 mM LaB and 5 mM fMLF to induce degranulation before assessment of surface expression of CD66b and CD63 by flow cytometry (*n* = 3 independent experiments). (**E**–**H**) Murine PMNs were exposed to 500 nM CD45 inhibitor VI, vehicle control (Vehicle), or HBSS^+^ (Ctrl) for 30 minutes at 37°C followed by stimulation with 1.25 mM LaB and 5 mM fMLF to induce degranulation before assessment of surface expression of CD66b and CD15 by flow cytometry. As a control, PMNs incubated with only HBSS+ were included (Ntx) (*n* = 3 independent experiments). Data in **C**, **D**, **G**, and **H** are the fold change in mean fluorescence intensity compared with unstimulated PMNs (Ctrl). Data are shown as mean ± SEM and were analyzed by 1-way ANOVA with Tukey’s post hoc testing. *P < 0.05; **P < 0.01; ***P < 0.001; ****P < 0.0001.

Given potent changes observed for PMN TEpM upon inhibition of CD45, the role of CD45 in regulating other critical PMN antimicrobial effector functions was assessed. We first evaluated consequences of inhibition of CD45 phosphatase activity on PMN degranulation in response to the potent stimuli Latrunculin B (LaB) combined with the formylated bacterial peptide fMLF. As expected, incubation with 1.25 mM LaB followed by 5 mM fMLF resulted in degranulation, as evidenced by increased surface expression of markers of primary (CD63) and secondary granules (CD66b) detected on the surface of human PMNs (Figure 2, A and B). Importantly, coincubation of PMNs with 500 nM CD45 inhibitor VI (but not a vehicle control) significantly reduced LaB- and fMLF-induced degranulation responses (Figure 2, C and D, *P* < 0.05 and *P* < 0.01). Analogous to results with human PMNs, significant LaB- and fMLF-mediated increases in surface expression of primary (CD63) and secondary (CD15) granules were observed in murine PMNs isolated from bone marrow (Figure 2, E and F). Furthermore, inhibition of CD45 phosphatase activity resulted in decreased surface expression of primary (CD63) and secondary granule (CD15) markers in LaB/fMLF-activated murine PMNs (Figure 2, G and H, *P* < 0.05, *P* < 0.01, *P* < 0.001, and *P* < 0.0001).

**Figure 3:**
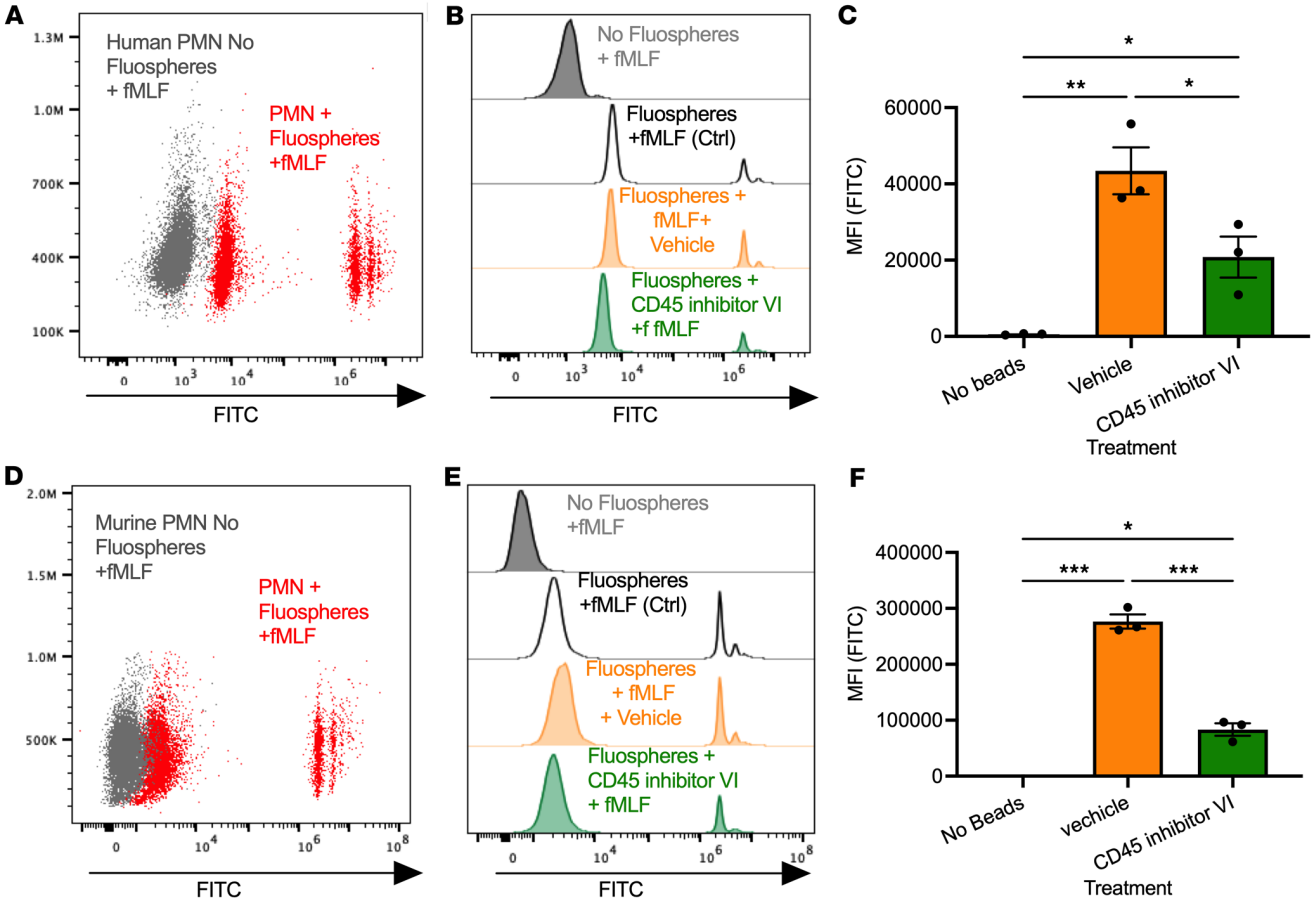
CD45 inhibition reduces phagocytosis by human and murine PMNs. (**A**–**C**) Human PMNs were incubated with 500 nM CD45 inhibitor VI, vehicle control (Vehicle), or Hanks balanced salt solution with Ca^2+^ and Mg^2+^ (HBSS^+^) (Ctrl) in conjunction with 100 nM fMLF for 60 minutes at 37°C before fluorescent microsphere phagocytosis/uptake was quantified by measuring changes in total levels of FITC fluorescence by flow cytometry (*n* = 3 independent experiments). (**D**–**F**) Murine PMNs were incubated with 500 nM CD45 inhibitor VI, vehicle control, or HBSS^+^ (Ctrl PMN) in conjunction with 200 nM fMLF for 60 minutes at 37°C before fluorescent microsphere phagocytosis/uptake was quantified by measuring changes in total levels of FITC fluorescence by flow cytometry (*n* = 3 independent experiments). Data in **C** and **F** are FITC mean fluorescence intensity (MFI) ± SEM and were analyzed by 1-way ANOVA with Tukey’s post hoc testing. *P < 0.05; **P < 0.01; ***P < 0.001.

In addition to degranulation, phagocytosis represents another critical effector function in the PMN antimicrobial arsenal that is essential for preventing infection and sterilizing repairing mucosal tissues. Therefore, we determined effects of CD45 inhibition on PMN engulfment of FluoSphere beads. As can be seen in Figure 3A, phagocytosis of FITC-positive beads by PMNs was readily detected by flow cytometry. Furthermore, analyses demonstrated that exposure of human PMNs to 500 nM CD45 inhibitor VI significantly reduced phagocytosis of FluoSphere beads relative to PMNs exposed to vehicle control (Figure 3, B and C, *P* < 0.05 and *P* < 0.01. A similar decrease in bead uptake was observed for murine PMNs exposed to CD45 inhibitor VI relative to vehicle control (Figure 3, D–F, *P* < 0.05 and *P* < 0.001). Taken together, these data suggest that CD45 phosphatase activity positively regulates degranulation and phagocytosis responses in both human and murine PMNs.

### Knockdown of CD45 regulates critical effector functions in human PMNs.

**Figure 4:**
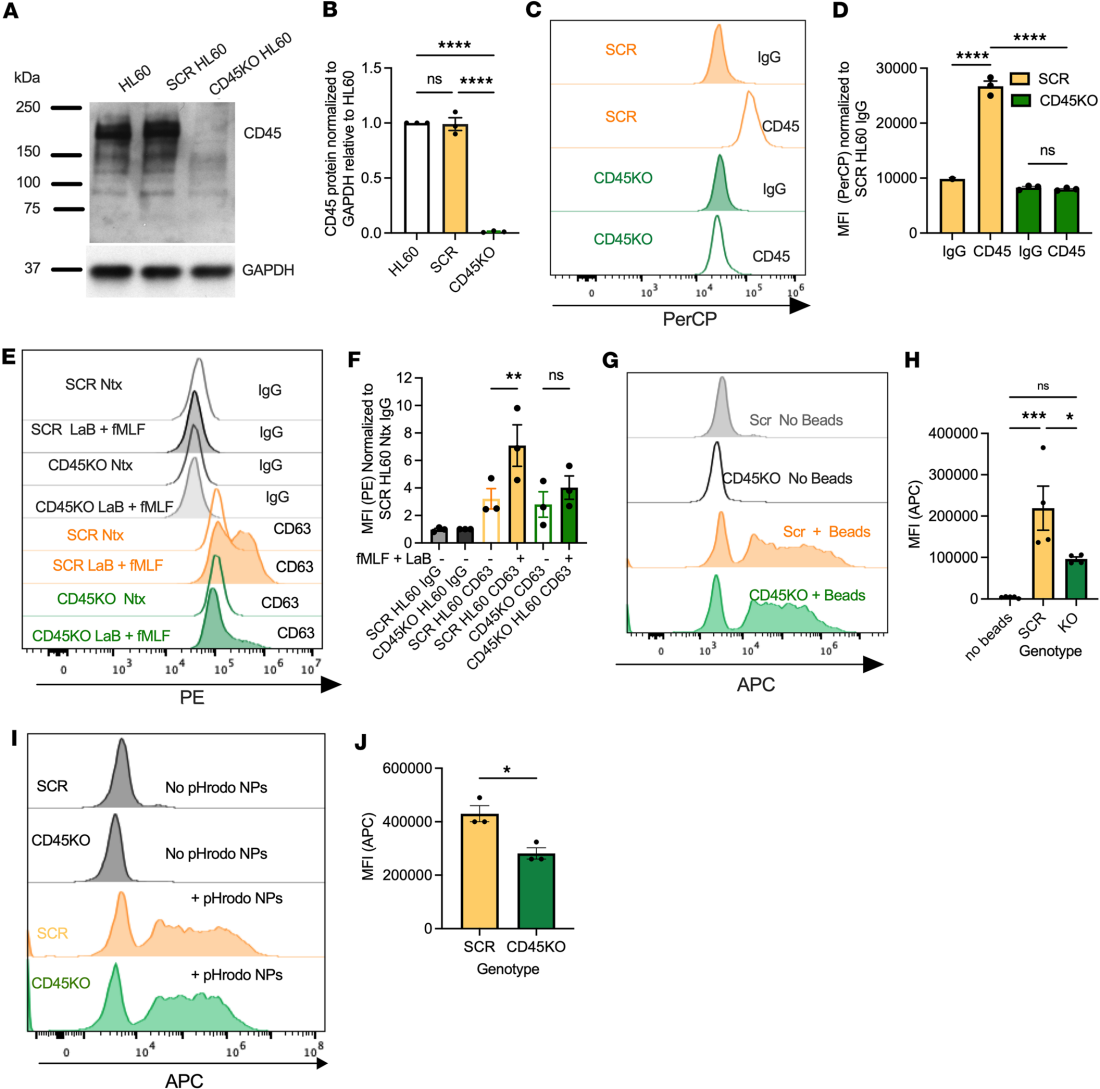
Knockout of CD45 in PMN-like HL60 cells reduces degranulation and phagocytosis. CD45 was knocked out by CRISPR/Cas9 in the human promyelocytic cell line HL60 (CD45KO). (**A**) Lysates from HL60, SCR HL60, or CD45KO HL60 cells differentiated into a PMN- like state were immunoblotted for CD45 or GAPDH. (**B**) Densitometry revealed a less than 98% reduction in CD45 expression (normalized to GAPDH) in CD45KO HL60 cells relative to non-transduced HL60 cells or SCR HL60 cells. Data were normalized to GAPDH and relative to non-transduced HL60 cells (*n* = 3 independent experiments). (**C** and **D**) Flow cytometric analysis using a PerCP-labeled anti-CD45 mAb and a PerCP-labeled IgG matched control mAb. Data represent mean fluorescence intensity (MFI) normalized to HL60 SCR IgG control (*n* = 3 independent experiments). (**E** and **F**) Differentiated SCR HL60 cells and CD45KO HL60 cells were stimulated with 1.25 mM LaB and 5 mM fMLF to induce degranulation. Data are the fold change in MFI for CD63 normalized to SCR HL60 IgG (*n* = 3 independent experiments). (**G** and **H**) Differentiated SCR HL60 and CD45KO HL60 cells were stimulated with 100 nM fMLF for 60 minutes at 37°C before fluorescent microsphere phagocytosis/uptake was quantified by measuring changes in total levels of APC fluorescence by flow cytometry. Data represent APC MFI (*n* = 4 independent experiments). (**I** and **J**) Differentiated CD45KO HL60 or SCR HL60 cells were incubated with 20 mg pHrodo-conjugated *E*. *coli* bioparticles in the presence of 10 nM fMLF. Phagocytosis was quantified by measuring changes in APC fluorescence by flow cytometry. Data are MFI values (*n* = 3 independent experiments). Data are shown as mean ± SEM and were analyzed by 1-way ANOVA with Tukey’s post hoc testing (**B**, **D**, **F**, **H**)or unpaired, 2-tailed *t* test (**J**). *P < 0.05; **P < 0.01; ***P < 0.001; ****P < 0.0001.

Given the potent functional effects observed upon inhibition of CD45 phosphatase activity, we generated human PMN-like promyelocytic HL60 cells lacking expression of CD45 (along with SCR control HL60 cells) using CRISPR/Cas9 editing. The human promyelocytic leukemia HL60 cell line was developed as a model system to study human PMN function. Importantly, DMSO-differentiated HL60 cells are a well-accepted system for modeling disease-relevant PMN functions, including oxidative burst, adhesion, chemotaxis, and TEpM (17). For all assays, HL60 cells were differentiated into a PMN-like phenotype using a modified version of a previously described technique (17). Western blotting analyses revealed a greater than 97% decrease in CD45 expression in CD45-KO (CD45KO) HL60 cells compared with non-transduced HL60 cells or SCR control HL60 cells (Figure 4, A and B, *P* < 0.0001). Flow cytometric analysis of CD45KO and SCR HL60 cells confirmed a total lack of CD45 surface expression on CD45KO HL60 cells (*P* < 0.0001, Figure 4, C and D). Consistent with inhibitor studies on human PMNs, CD45KO HL60 cells were found to have reduced degranulation/CD63 surface expression in response to LaB/fMLF stimulation relative to SCR HL60 cells (*P* < 0.01, Figure 4, E and F). Similarly, significantly decreased phagocytosis of FluoSpheres by CD45KO HL60 cells relative to SCR HL60 cells was observed (Figure 4, G and H, *P* < 0.05 and *P* < 0.001). To verify intracellular uptake of fluorescent microspheres, CD45KO HL60 and CD45 SCR HL60 cells were incubated with pHrodo-conjugated *E*. *coli* bioparticles that emit no fluorescent signal at neutral pH but fluoresce brightly in intracellular acidic environments. Importantly, significantly decreased phagocytosis of pHrodo-conjugated *E*. *coli* was observed in CD45KO HL60 cells relative to SCR HL60 cells (Figure 4, I and J, *P* < 0.05). As expected, significantly less phagocytosis of pHrodo-conjugated *E*. *coli* was observed in PMN-like HL60 cells incubated at 37°C relative to 4°C (Supplemental Figure 2, A and B, *P* < 0.05). Taken together, these data show that inhibition of CD45 phosphatase activity or KO of CD45 expression results in downregulation of critical PMN functional responses.

### Generation of PMN-specific CD45 knockdown mice.

**Figure 5:**
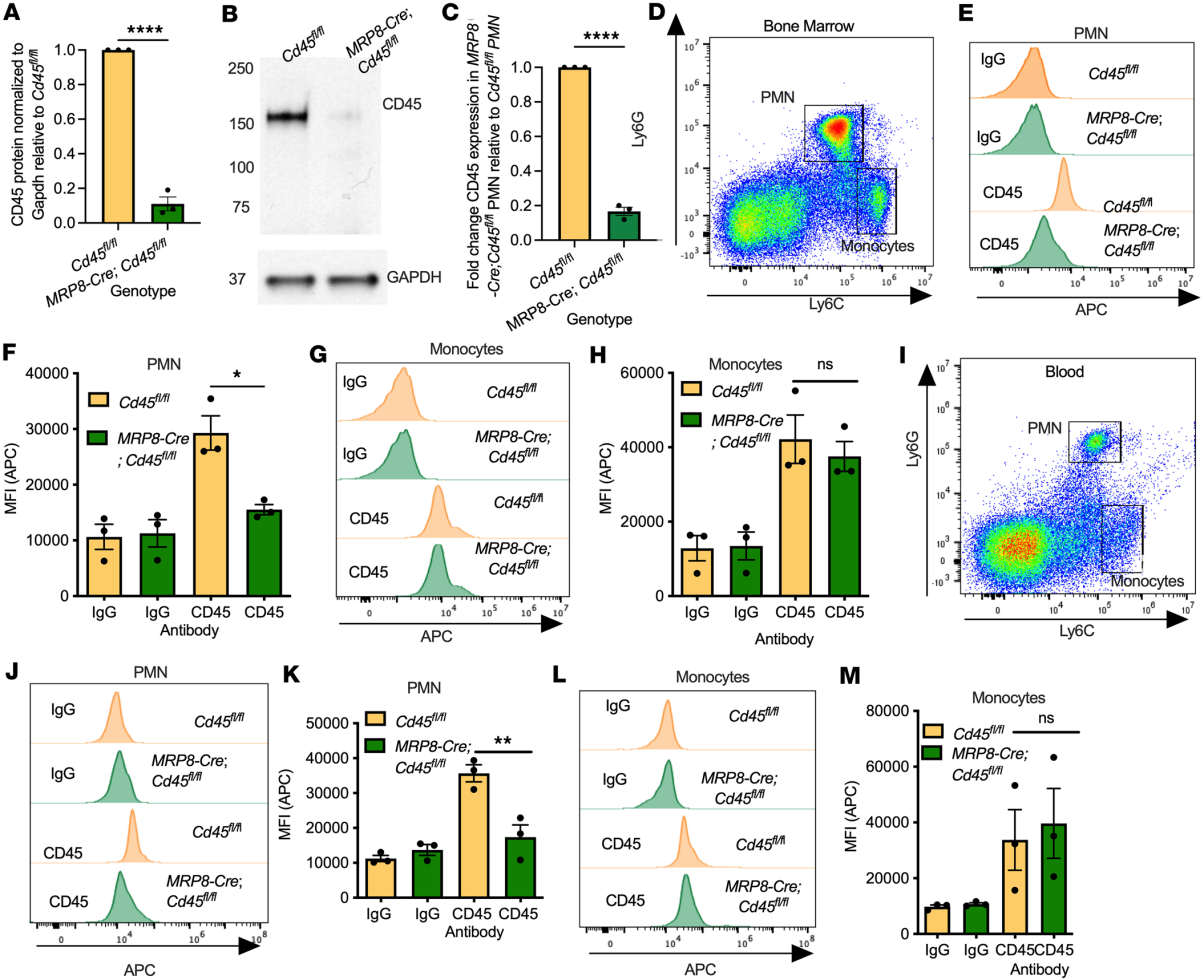
Generation of PMN-specific CD45-deficient mice. (**A**) PCR revealed a 90% reduction in *Cd45* expression in PMNs from *MRP8-Cre;Cd45^fl/fl^* mice compared with *Cd45^fl/fl^* PMNs (*n* = 3 independent experiments). (**B**) Representative immunoblot showing CD45 expression in *MRP8-Cre;Cd45^fl/fl^* versus *Cd45^fl/fl^* PMNs. Data represent PMNs isolated from 3 mice per genotype. (**C**) Densitometry revealed a 90% reduction in CD45 expression in *MRP8-Cre;Cd45^fl/fl^* PMNs relative to *Cd45^fl/fl^* PMNs. Data represent mean band intensity normalized to GAPDH relative to *Cd45^fl/fl^* (*n* = 3 independent experiments). (**D**) Gating strategy for bone marrow immune cells showing CD11b^+^Siglec-F^–^Ly6G^+^Ly6C^+^ cells (PMNs) and CD11b^+^Siglec-F^–^Ly6G^–^Ly6C^+^ cells (monocytes). (**E** and **F**) Flow cytometric analysis of Ly6G^+^Ly6C^+^ PMNs shows a 90% reduction in CD45 surface expression in bone marrow *MRP8-Cre;Cd45^fl/fl^* PMNs compared with *Cd45^fl/fl^* PMNs. Data represent mean fluorescence intensity (MFI) (*n* = 3 independent experiments). (**G** and **H**) Flow cytometric analysis of CD45 surface expression in bone marrow monocytes (Ly6G^–^Ly6C^+^) from *MRP8-Cre;Cd45^fl/fl^* mice compared with *Cd45^fl/fl^* control mice. Data represent MFI (*n* = 3 independent experiments). (**I**) Gating strategy for circulating immune cells showing CD11b^+^Siglec-F^–^Ly6G^+^Ly6C^+^ cells (PMNs) and CD11b^+^Siglec-F^–^Ly6G^–^Ly6C^+^ cells (monocytes). (**J** and **K**) Flow cytometric analysis of Ly6G^+^Ly6C^+^ PMNs shows a 90% reduction in CD45 surface expression in circulating PMNs (Ly6G^+^Ly6C^+^) from *MRP8-Cre;Cd45^fl/fl^* mice compared with *Cd45^fl/fl^* control mice. Data represent MFI (*n* = 3 independent biological experiments). (**L** and **M**) Flow cytometry shows unchanged CD45 surface expression in monocytes (Ly6G^–^Ly6C^+^) isolated from blood of *MRP8-Cre;Cd45^fl/fl^* mice relative to *Cd45^fl/fl^* control mice. Data represent MFI (*n* = 3 independent experiments). Data are shown as mean ± SEM and were analyzed by 1-way ANOVA with Tukey’s post hoc testing (**F**, **H**, **K**, **M**)or unpaired, 2-tailed *t* test (**A**, **C**). *P < 0.05; **P < 0.01; ****P < 0.0001.

CD45 is expressed on all leukocytes, and mice with global KO of *Cd45* have traditionally been used to study the role of CD45 in T and B cell function. Such mice exhibit numerous immune deficiencies, including defective thymocyte maturation, reduced peripheral T cell numbers, and impaired B cell proliferation (18, 19). Therefore, to better understand the specific role of CD45 in regulating PMN function during intestinal inflammation and repair in the absence of adaptive immune irregularities, we generated what are believe to be novel PMN-targeted *Cd45*-deficient mice. For this, *Cd45^fl/fl^* mice were crossed with mice expressing Cre recombinase under the granulocyte-specific promoter MRP8 (*MRP8-Cre-IRES/GFP*) to specifically excise CD45 in PMNs (*MRP8-Cre;Cd45^fl/fl^*) (Supplemental Figure 3, A and B). *MRP8-Cre;Cd45^fl/fl^* mice developed normally and exhibited no differences in size or anatomical features compared to floxed littermate controls, with no baseline intestinal pathology observed (Supplemental Figure 4, A and B). Furthermore, quantification of numbers of PMNs (Siglec-F^+^CD11b^+^Ly6G^+^Ly6C^+^), monocytes (Siglec-F^+^CD11b^+^Ly6G^+^Ly6C^+^), eosinophils (Siglec-F^+^CD11b^+^), B cells (CD19^+^CD3^–^), and T cells (CD4^+^CD3^+^) in the blood and bone marrow of *MRP8-Cre;Cd45^fl/fl^* mice revealed no changes relative to *Cd45^fl/fl^* control mice (Supplemental Figure 5). PCR of bone marrow PMNs revealed an 85% or greater reduction in *Cd45* expression in *MRP8-Cre;Cd45^fl/fl^* mice relative to *Cd45^fl/fl^* control mice (Figure 5A, *P* < 0.0001). Immunoblotting and densitometry analyses confirmed an 85% or greater reduction in CD45 protein expression in bone marrow–derived PMNs from *MRP8-Cre;Cd45^fl/fl^* mice (Figure 5, B and C, *P* < 0.0001). Flow cytometric quantification confirmed an 85% or greater decrease in surface expression of CD45 on bone marrow–derived PMNs (Ly6G^+^Ly6C^+^) from MRP8-*Cre*;*Cd45^fl/fl^* mice compared with *Cd45^fl/fl^* control mice (Figure 5, D–F, *P* < 0.05). In contrast, no change in surface expression of CD45 was observed for bone marrow monocytes from MRP8-*Cre*;*Cd45^fl/fl^* mice compared to *Cd45^fl/fl^* control mice (Figure 5, G and H). As was observed for immune cells isolated from bone marrow, a statistically significant decrease in CD45 surface expression was observed for circulating PMNs isolated from blood of MRP8-*Cre*;*Cd45^fl/fl^* mice relative to *Cd45^fl/fl^* control mice (Figure 5, I–K, *P* < 0.01). Importantly, blood monocytes from MRP8-*Cre*;*Cd45^fl/fl^* mice also showed no reduction in CD45 surface expression (Figure 5, L and M).

Given the adaptive immune irregularities reported for total CD45KO mice (18, 19), CD45 expression in other relevant innate and adaptive immune cells was assessed. Importantly no change in CD45 surface expression was observed for B cells (CD19^+^CD3^–^), T cells (CD4^+^CD3^+^), or eosinophils (Siglec-F^+^CD11b^+^) isolated from bone marrow of *MRP8-Cre;Cd45^fl/fl^* mice relative to *Cd45^fl/fl^* control mice (Supplemental Figure 6). Furthermore, no change in CD45 surface expression was observed on B cells (CD19^+^CD3^–^), T cells (CD4^+^CD3^+^), or eosinophils (Siglec-F^+^CD11b^+^) isolated from blood of *MRP8-Cre;Cd45^fl/fl^* mice relative to *Cd45^fl/fl^* control mice (Supplemental Figure 7). Taken together, these data demonstrate generation of mice with PMN-specific knockdown of *Cd45* expression.

### CD45 depletion downregulates critical PMN effector functions.

**Figure 6:**
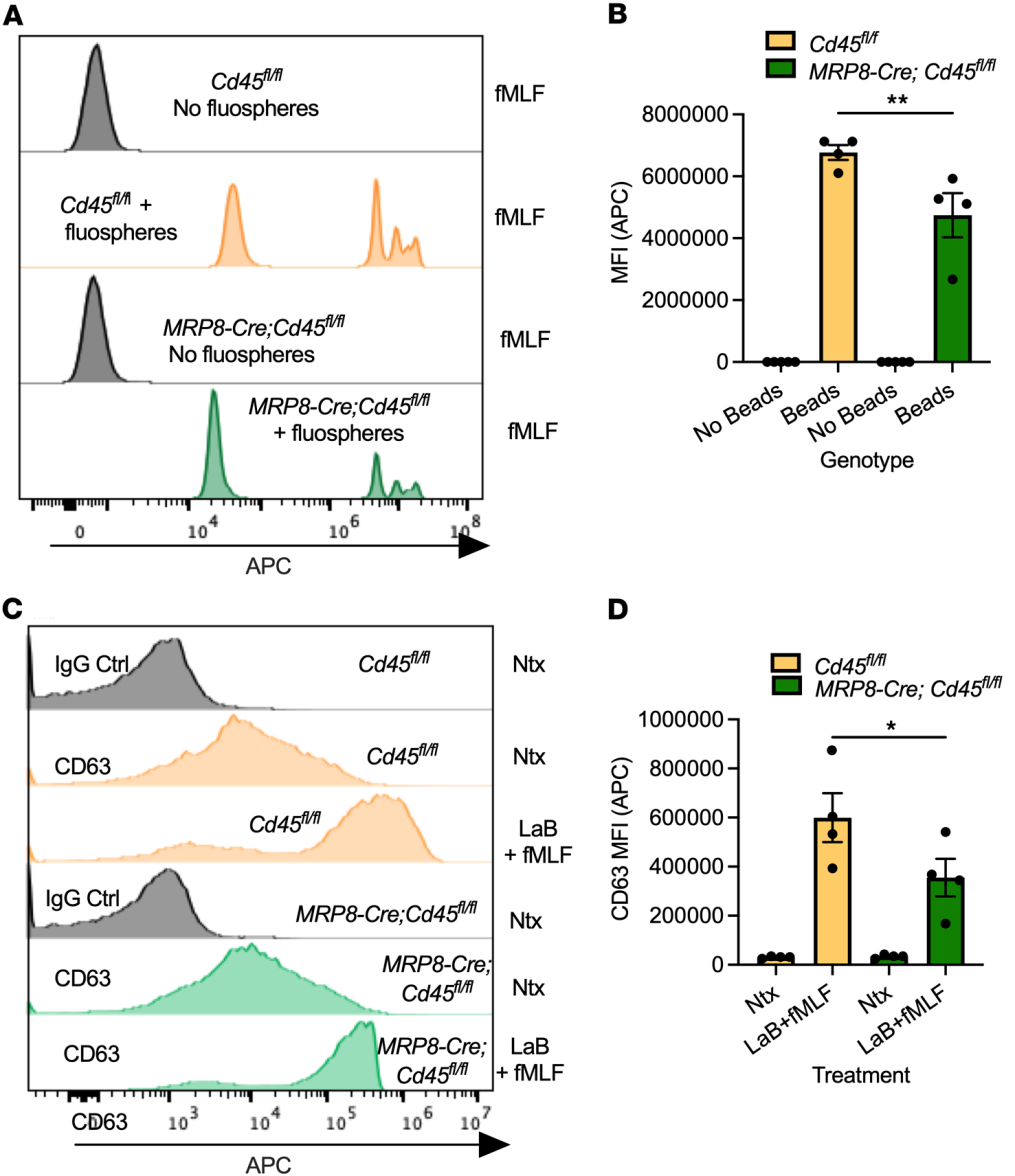
*Cd45* depletion decreases PMN degranulation and phagocytosis responses. (**A** and **B**) PMNs isolated from *MRP8-Cre;Cd45^fl/fl^* mice and control *Cd45^fl/fl^* mice were stimulated with 100 nM fMLF for 60 minutes at 37°C before fluorescent microsphere phagocytosis/uptake was quantified by flow cytometry. Data represent fold change in total APC mean fluorescence intensity (MFI) (*n* = 4 independent biological experiments). (**C** and **D**) PMNs isolated from *MRP8-Cre;Cd45^fl/fl^* mice and control *Cd45^fl/fl^* mice were stimulated with 1.25 mM LaB and 5 mM fMLF to induce degranulation before assessment of surface expression of CD63 by flow cytometry. Data represent APC MFI (*n* = 4 independent experiments). Data are shown as mean ± SEM and were analyzed by 1-way ANOVA with Tukey’s post hoc testing. *P < 0.05; **P < 0.01.

Given data showing decreased TEpM and antimicrobial function in PMNs upon CD45 inhibition, key functional outputs were analyzed in PMNs isolated from *MRP8-Cre;Cd45^fl/fl^* mice. Figure 6, A and B demonstrates significantly decreased phagocytosis of FluoSpheres by bone marrow PMNs isolated from *MRP8-Cre;Cd45^fl/fl^* mice relative to PMNs from *Cd45^fl/fl^* mice (*P* < 0.01). Similarly, a significant decrease in LaB/fMLF-induced degranulation of primary granules was observed for PMNs from *MRP8-Cre;Cd45^fl/fl^* mice relative to PMNs from *Cd45^fl/fl^* control mice (Figure 6, C and D, *P* < 0.05). Taken together, the data from PMN-specific *Cd45*-deficient mice confirm an important regulatory role for CD45 in driving effector functions of activated PMNs.

### PMN-specific knockdown of Cd45 delays recovery from DSS-induced colitis and reduces PMN intestinal trafficking.

**Figure 7:**
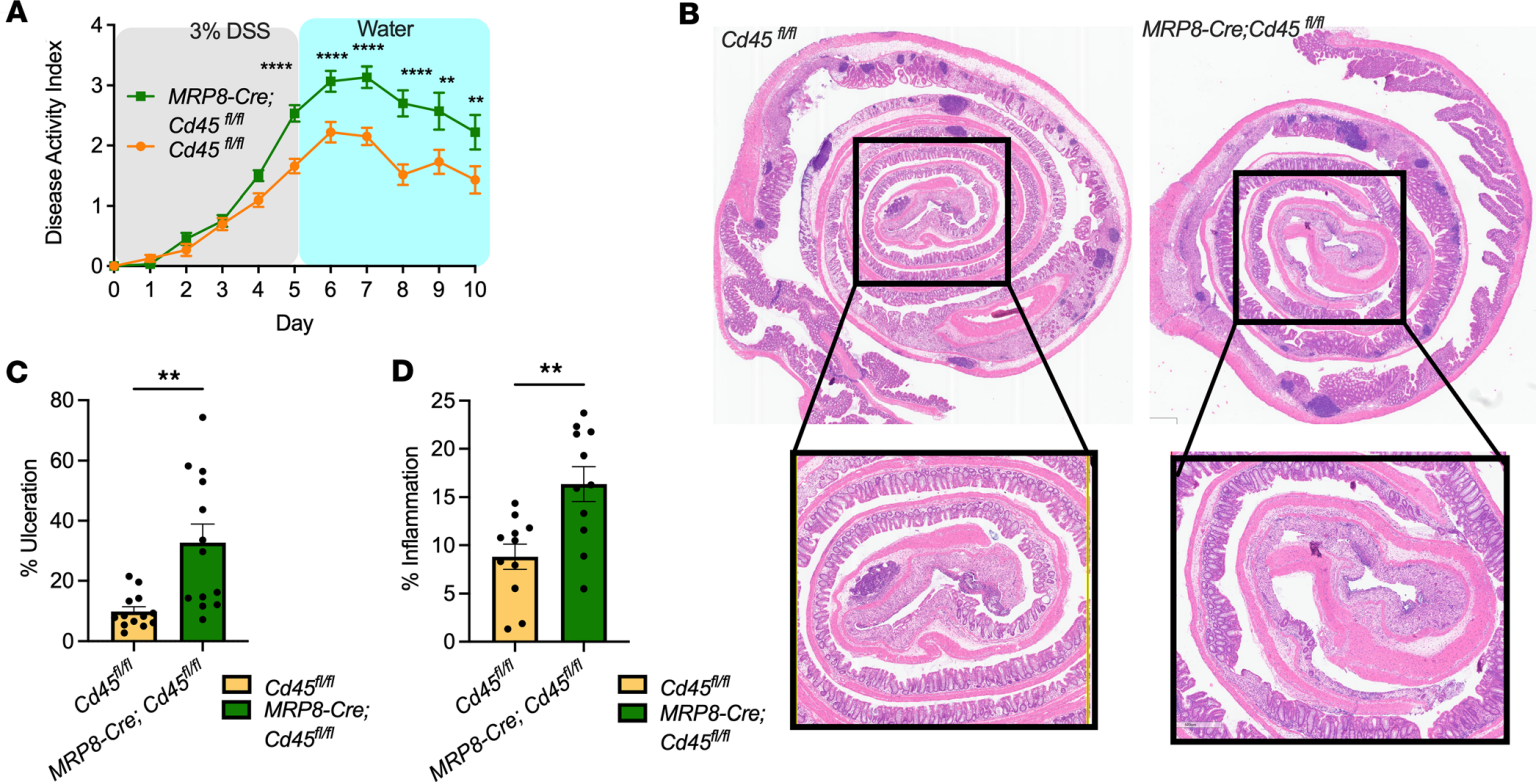
Knockdown of *Cd45* in PMNs delays recovery from DSS colitis. (**A**) Disease Activity Index (DAI) for *MRP8-Cre;Cd45^fl/fl^* mice and *Cd45^fl/fl^* mice administered water containing 3% DSS for 5 days followed by regular water for 5 days (*n* = 3 independent experiments with 5–10 mice per group). (**B**) On day 10, mice were euthanized and tissues harvested for histological analysis. Representative histological images of Swiss rolls of whole colons with magnifications of the rectum to assess levels of DSS-induced colitis. Left image is from a *Cd45^fl/fl^* mouse, right image is from an *MRP8-Cre;Cd45^fl/fl^* mouse. (**C** and **D**) Histological scoring of colonic tissue on day 10 evaluated by 2 independent investigators (each dot represents data from an individual mouse across 2 independent experiments, 13 mice per group). Data are shown as mean ± SEM and were analyzed by 1-way ANOVA with Tukey’s post hoc testing (**A**) or unpaired, 2-tailed *t* test (**C**, **D**). **P < 0.01; ****P < 0.0001.

Having established that *MRP8-Cre;Cd45^fl/fl^* mice do not develop any intestinal pathology under baseline conditions and have unchanged numbers of immune cells in circulation and in the bone marrow (Supplemental Figure 5), we next assessed effects of PMN-specific *Cd45* depletion on intestinal mucosal repair. When subjected to a cycle of DSS administration followed by water recovery, *MRP8-Cre;Cd45^fl/fl^* mice exhibited significantly increased disease activity scores (aggregate scores encompassing body weight, stool consistency, and occult blood levels) on days 5–10 compared with *Cd45^fl/fl^* control mice (Figure 7A, *P* < 0.01 and *P* < 0.0001). These data indicate decreased mucosal healing in the gut in the absence of CD45-expressing PMNs. Histological scoring revealed a significant increase in the percentage of ulceration and inflammation in *MRP8-Cre;Cd45^fl/fl^* mice relative to *Cd45^fl/fl^* control mice on day 10 (Figure 7, B–D, *P* < 0.01). Analyses of lamina propria cell contents on day 8 (day 3 of repair phase) revealed a significant decrease in both luminal PMNs and PMN associating with colonic IECs in *MRP8-Cre;Cd45^fl/fl^* mice, confirming a defect in the ability of CD45-deficient PMNs to undergo TEpM (Supplemental Figure 8, A and B, *P* < 0.05 and *P* < 0.01). Taken together, these data suggest that mice with PMNs deficient in CD45 exhibit decreased PMN trafficking to the intestine along with delayed mucosal repair following DSS-induced colitis.

### CD45 depletion in PMNs reduces healing of biopsy-induced mucosal colonic wounds in vivo.

**Figure 8:**
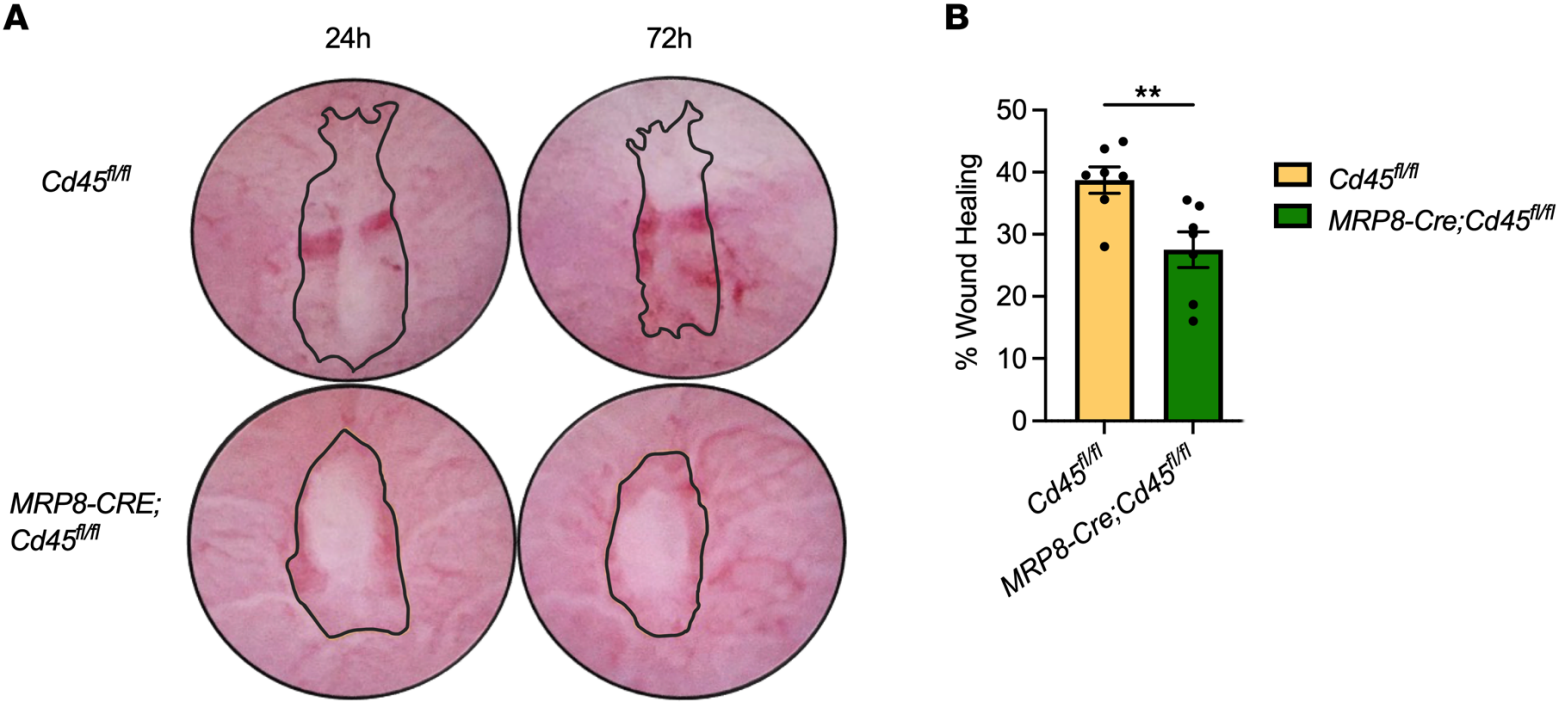
PMN-specific depletion of *Cd45* delays healing of biopsy-induced colonic wounds in vivo. (**A**) Representative images of wounds from *MRP8-Cre;Cd45^fl/fl^* and *Cd45^fl/fl^* mice at 24 and 72 hours after biopsy wounding. (**B**) Quantification of percentage wound healing in *MRP8-Cre;Cd45^fl/fl^* and *Cd45^fl/fl^* mice at 72 hours after biopsy wounding. Each dot represents data from 1 mouse with an average of 4–6 wounds per mouse; *n* = 2 independent experiments. Data are shown as mean ± SEM and were analyzed by unpaired, 2-tailed *t* test. **P < 0.001.

Given potent effects on recovery from DSS-induced colitis, we next examined effects of PMN-specific CD45 depletion on colonic repair following in vivo biopsy wounding (15). As can be seen in Figure 8, PMN-specific depletion of CD45 in *MRP8-Cre;Cd45^fl/fl^* mice resulted in significantly decreased rates of colonic mucosal wound repair between 24 and 72 hours after wounding relative to *Cd45^fl/fl^* control mice (Figure 8, A and B, *P* < 0.01). Taken together, these data demonstrate that signaling through PMN-expressed CD45 positively regulates mucosal wound repair following chemical or mechanical insult in vivo.

### CD45 depletion or inhibition decreases PMN CD11b/CD18 surface expression and activation.

**Figure 9:**
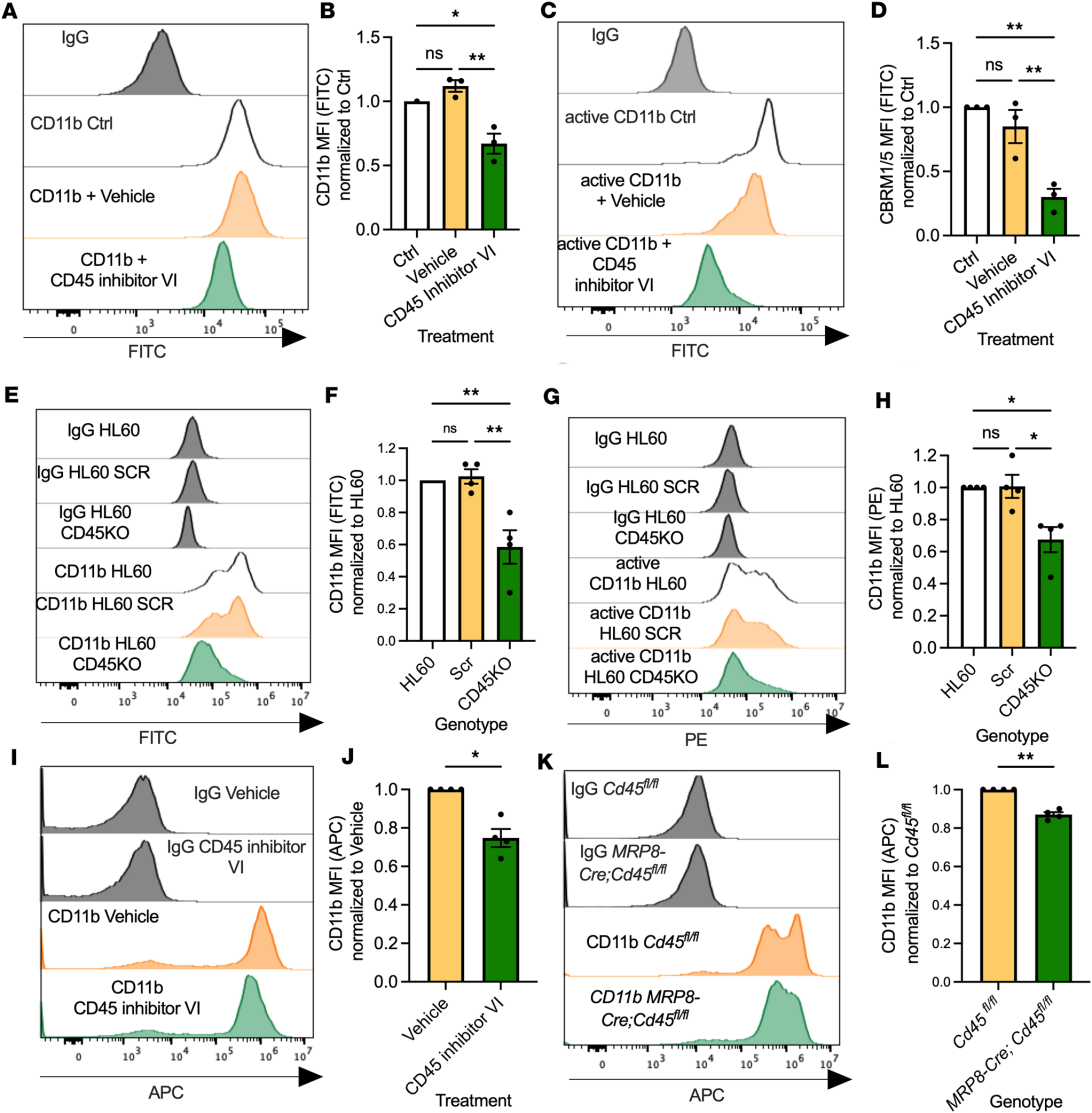
Inhibition or depletion of CD45 reduces CD11b/CD18 surface expression and activation on PMNs. (**A** and **B**) Human PMNs exposed to 500 nM CD45 inhibitor VI or vehicle control were incubated with a FITC-conjugated anti-CD11b mAb or isotype control mAb and assessed by flow cytometry. Data represent fold change in CD11b mean fluorescence intensity (MFI) normalized to Ctrl (*n* = 3 independent biological experiments). (**C** and **D**) Human PMNs exposed to 500 nM CD45 inhibitor VI or vehicle control were assessed for CD11b activation by flow cytometry. Data represent fold change in CBRM1/5 MFI normalized to Ctrl (*n* = 3 independent experiments). (**E** and **F**) CD11b surface expression detected by flow cytometry of CD45KO HL60, SCR HL60, or non-transduced HL60 cells. Data represent fold change in CD11b MFI normalized to non-transduced HL60 cells (*n* = 3 independent experiments). (**G** and **H**) Surface expression of active CD11b in CD45KO HL60, SCR HL60, or non-transduced HL60 cells detected by flow cytometry. Data represent fold change in CBRM1/5 MFI normalized to non-transduced HL60 cells (*n* = 3 independent experiments). (**I** and **J**) WT murine PMNs were exposed to 500 nM CD45 inhibitor VI or vehicle control and incubated with an APC-conjugated anti-CD11b mAb or an isotype control mAb before flow cytometric analysis of CD11b surface expression. Data represent fold change in CD11b MFI normalized to vehicle control (*n* = 4 independent experiments). (**K** and **L**) PMNs from *MRP8-Cre;Cd45^fl/fl^* mice or *Cd45^fl/fl^* mice were incubated with an APC-conjugated anti-CD11b mAb or isotype control mAb before analysis of CD11b surface expression by flow cytometry. Data represent fold change in CD11b MFI normalized to *Cd45^fl/fl^* PMNs (*n* = 4 independent experiments). Data represent mean ± SEM and were analyzed by 1-way ANOVA with Tukey’s post hoc testing (**B**, **D**, **F**, **H**) or unpaired, 2-tailed *t* test (**J**, **L**). *P < 0.05; **P < 0.01.

Previous studies have implicated the β2 integrin CD11b/CD18 in playing a role in regulating PMN migration and other important effector functions via incompletely understood molecular mechanisms (9–11, 20, 21). We therefore investigated effects of knockdown or inhibition of CD45 on PMN CD11b expression and activation. Flow cytometric analysis revealed that inhibition of CD45 in human PMNs with 500 nM CD45 inhibitor VI significantly reduced surface expression of CD11b relative to vehicle-treated PMNs (Figure 9, A and B, *P* < 0.05 and *P* < 0.01). Given that activation-dependent conformational changes in CD11b/CD18 facilitate enhanced ligand binding and downstream signaling (22), we probed the effect of CD45 inhibition on CD11b/CD18 activation using a mAb that specifically recognizes an activation epitope on human CD11b (CBRM1/5). Indeed, inhibition of CD45 resulted in significantly reduced levels of active CD11b/CD18 on the surface of human PMNs (Figure 9, C and D, *P* < 0.01). Furthermore, flow cytometric analyses of CD45KO PMN-like HL60 cells confirmed decreased CD11b surface expression and activation in the absence of CD45 expression (Figure 9, E–H, *P* < 0.05 and *P* < 0.01).

As was observed for human PMNs, exposure of murine PMNs to 500 nm CD45 inhibitor VI significantly reduced surface expression of CD11b relative to vehicle-treated PMNs (Figure 9, I and J, *P* < 0.05). Finally, comparison of PMNs from *MRP8-Cre;Cd45^fl/fl^* mice to PMNs from *Cd45^fl/fl^* mice revealed a marked decrease in CD11b surface expression in the absence of CD45 expression (Figure 9, K and L, *P* < 0.01). In sum, these data suggest that functional effects observed upon CD45 inhibition or depletion may be in part mediated via downregulation of PMN CD11b/CD18 surface expression and activation.

### CD45 dephosphorylates Lyn kinase to regulate critical functional responses in PMNs.

**Figure 10:**
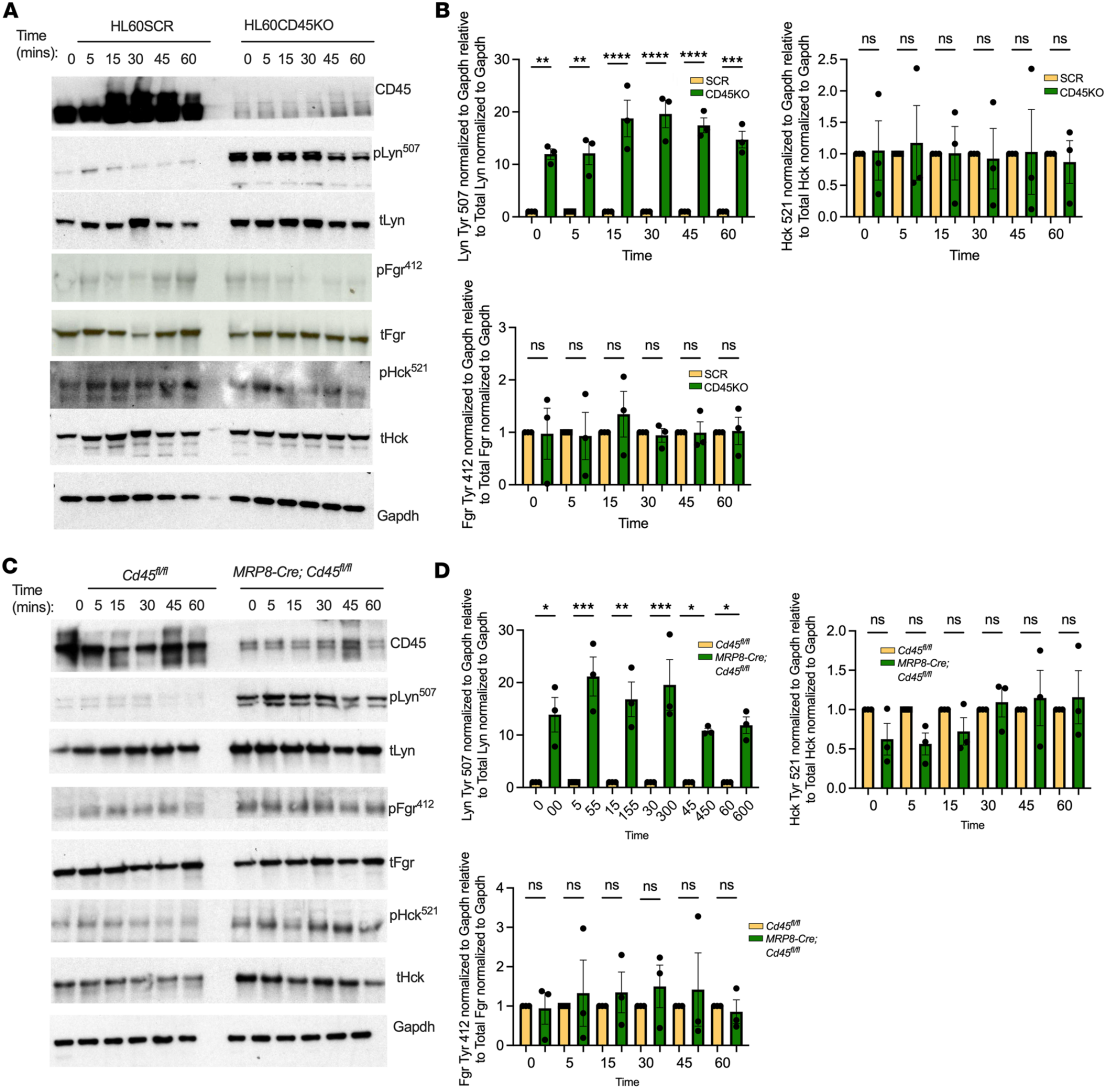
CD45 depletion results in constitutive Lyn kinase deactivation in PMNs. (**A**) Differentiated CD45KO HL60 cells or SCR control HL60 PMN-like cells were stimulated with 100 nM fMLF over a 1-hour time course. Representative blots (*n* = 3 independent experiments) are shown for indicated proteins or phosphoproteins. (**B**) Densitometry showing statistically significant decreases in Lyn dephosphorylation at Tyr507 but no changes in Hck or Fgr phosphorylation upon CD45 knockdown. Data represent phosphorylated protein levels normalized to GAPDH relative to total protein levels normalized to GAPDH (*n* = 3). (**C**) PMNs from *MRP8-Cre;Cd45^fl/fl^* mice and *Cd45^fl/fl^* mice were stimulated with 100 nM fMLF over a 1-hour time course. Representative blots are shown for indicated proteins. Blots are representative of *n* = 3 independent experiments. (**D**) Densitometry showing statistically significant decreases in Lyn dephosphorylation at Tyr507 but no changes in Hck or Fgr phosphorylation in PMNs from *MRP8-Cre;Cd45^fl/fl^* mice relative to PMNs from *Cd45^fl/fl^* mice. Data represent phosphorylated protein levels normalized to GAPDH relative to total protein levels normalized to GAPDH (*n* = 3). Data represent mean ± SEM and were analyzed by 1-way ANOVA with Tukey’s post hoc testing. *P < 0.05; **P < 0.01; ***P < 0.001; ****P < 0.0001.

While it is well established that CD45 regulates T and B cell function by dephosphorylating regulatory tyrosine residues on specific SFK members, including Lck and Fyn (7, 8), less is known about CD45-mediated intracellular signaling in PMNs. Therefore, we assessed effects of CD45 depletion on phosphorylation of the 3 PMN-expressed SFK members (Hck, Fgr, and Lyn) over a 60-minute time course of fMLF activation. As expected, robust knockdown of CD45 was observed in CD45-deficient HL60 cells (Figure 10A). Furthermore, there was significantly increased phosphorylation of Lyn at Tyr507 in CD45KO HL60 cells relative to CD45-expressing (SCR) HL60 cells. Densitometric analysis confirmed increased inhibitory phosphorylation of Lyn at Tyr507 in the absence of CD45 expression at all time points between 0 and 60 minutes (Figure 10B, *P* < 0.01, *P* < 0.001, and *P* < 0.0001) of fMLF activation. Importantly no significant difference in phosphorylation of other PMN-expressed SFKs (Hck and Fgr) was observed in CD45KO HL60 cells (Figure 10, A and B). In keeping with specific changes in Lyn activation in CD45-deficient human PMNs, increased inhibitory phosphorylation of Lyn at Tyr507 was observed in PMNs isolated from *MRP8-Cre;Cd45^fl/fl^* relative to *Cd45^fl/fl^* control mice (Figure 10C). Densitometric analysis confirmed significant increases in Lyn phosphorylation at Tyr507 at all time points measured between 0 and 60 minutes of fMLF stimulation in CD45-deficient murine PMNs (Figure 10D, *P* < 0.05, *P* < 0.01, and *P* < 0.001). Furthermore, no significant difference in phosphorylation of Hck or Fgr was observed in CD45-deficient murine PMNs (Figure 10D). Taken together, these data demonstrate robust and specific deactivation of Lyn kinase when CD45 is absent from PMNs, suggesting that CD45-mediated Lyn dephosphorylation at Tyr507 is a critical regulator of PMN effector functions.

**Figure 11:**
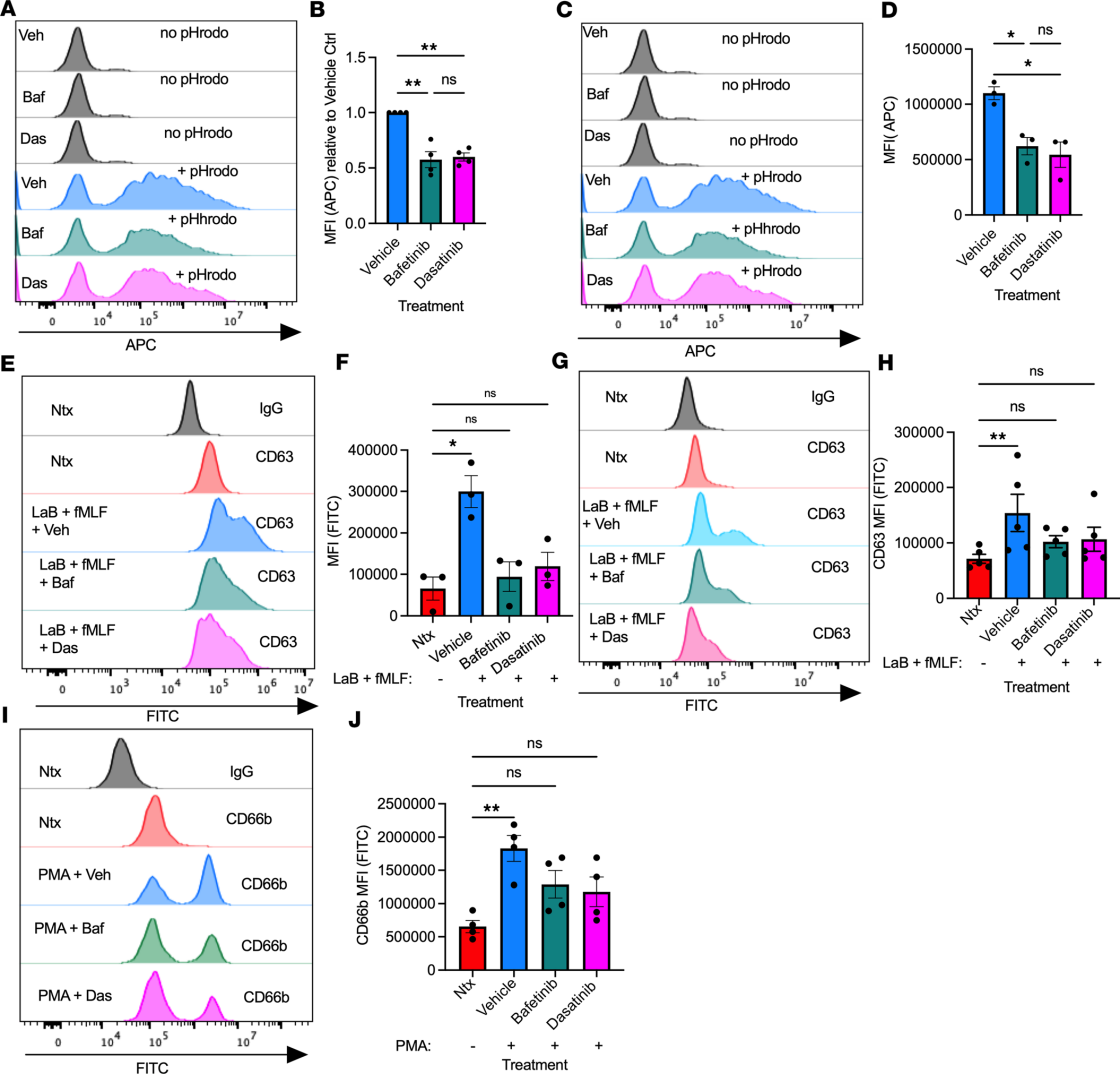
Inhibition of Lyn kinase with Bafetinib or Dasatinib mimics functional effects of CD45 inhibition or deletion. Differentiated HL60 cells in suspension (**A** and **B**) or adhering to fibronectin (**C** and **D**) were incubated with 100 nM Bafetinib, 250 nM Dasatinib, or vehicle control before addition of pHrodo-conjugated *E*. *coli* particles in the presence of 10 nM fMLF. Phagocytosis was quantified by measuring changes in APC fluorescence by flow cytometry. Data represent mean fluorescence intensity (MFI) values normalized to vehicle control (*n* = 3 independent experiments). PMN-like HL60 cells in suspension (**E** and **F**) or adhering to fibronectin (**G** and **H**) were incubated with 100 nM Bafetinib, 250 nM Dasatinib, or vehicle control before stimulation with 1.25 mM LaB and 5 mM fMLF to induce degranulation followed by assessment of surface expression of CD63 by flow cytometry. Data shown are FITC/CD63 MFI values (*n* = 5 independent experiments). (**I** and **J**) Human PMNs adhering to fibronectin were incubated with 100 nM Bafetinib, 250 nM Dasatinib, or vehicle control before stimulation with 100 nM PMA to induce degranulation followed by assessment of surface expression of CD66b by flow cytometry (*n* = 3 independent experiments). Data represent mean ± SEM and were analyzed by 1-way ANOVA with Tukey’s post hoc testing. *P < 0.05; **P < 0.01.

To verify that Lyn kinase is a key mediator downstream of CD45 in PMNs, the effects of two Lyn inhibitors (Bafetinib and Dasatinib) on PMN-like HL60 cell function were next assessed. Western blotting verified that at the concentrations used for functional assays, Dasatinib and Bafetinib did not significantly alter phosphorylation of additional PMN-expressed SFK members, Hgr and Fck (Supplemental Figure 9, A and B). Importantly, flow cytometric analysis revealed that incubation with 100 nM Bafetinib or 250 nM Dasatinib resulted in a significant decrease in phagocytosis of pHrodo-conjugated *E*. *coli* bioparticles in PMN-like differentiated HL60 cells (Figure 11, A and B, *P* < 0.01). To determine the effects of Lyn kinase inhibition on phagocytosis by adherent cells, PMN-like HL60 cells were allowed to adhere to fibronectin before incubation with 100 nM Bafetinib or 250 nM Dasatinib and pHrodo-conjugated *E*. *coli* bioparticles. As can be seen in Figure 11, C and D, inhibition of Lyn kinase in adherent PMN-like HL60 cells resulted in a significant decrease in phagocytosis of pHrodo-conjugated *E*. *coli* bioparticles (*P* < 0.05). The effects of Lyn kinase inhibition on degranulation were next assessed. Flow cytometric analyses revealed that incubation of PMN-like HL60 cells in suspension with 100 nM Bafetinib or 250 nM Dasatinib resulted in decreased LaB/fMLF-induced CD63 surface expression (Figure 11, E and F, *P* < 0.05). In addition, decreased LaB/fMLF-induced degranulation was also observed in differentiated HL60 cells adhering to fibronectin (Figure 11, G and H, *P* < 0.01). Finally, we showed that Lyn kinase inhibition reduced degranulation in human PMNs without the need for priming with LaB. As can be seen in Figure 11, I and J, treatment of adherent human PMNs exposed to vehicle control with 100 nM PMA resulted in a significant increase in the surface expression of CD66b (*P* < 0.01). However, exposure of adherent PMNs to Bafetinib or Dasatinib reduced degranulation/CD66b surface upregulation induced by PMA stimulation (Figure 11, I and J). Taken together, these data demonstrate that inhibition of Lyn kinase phenocopies inhibitory effects on PMN function observed when CD45 is inhibited or depleted.

**Figure 12:**
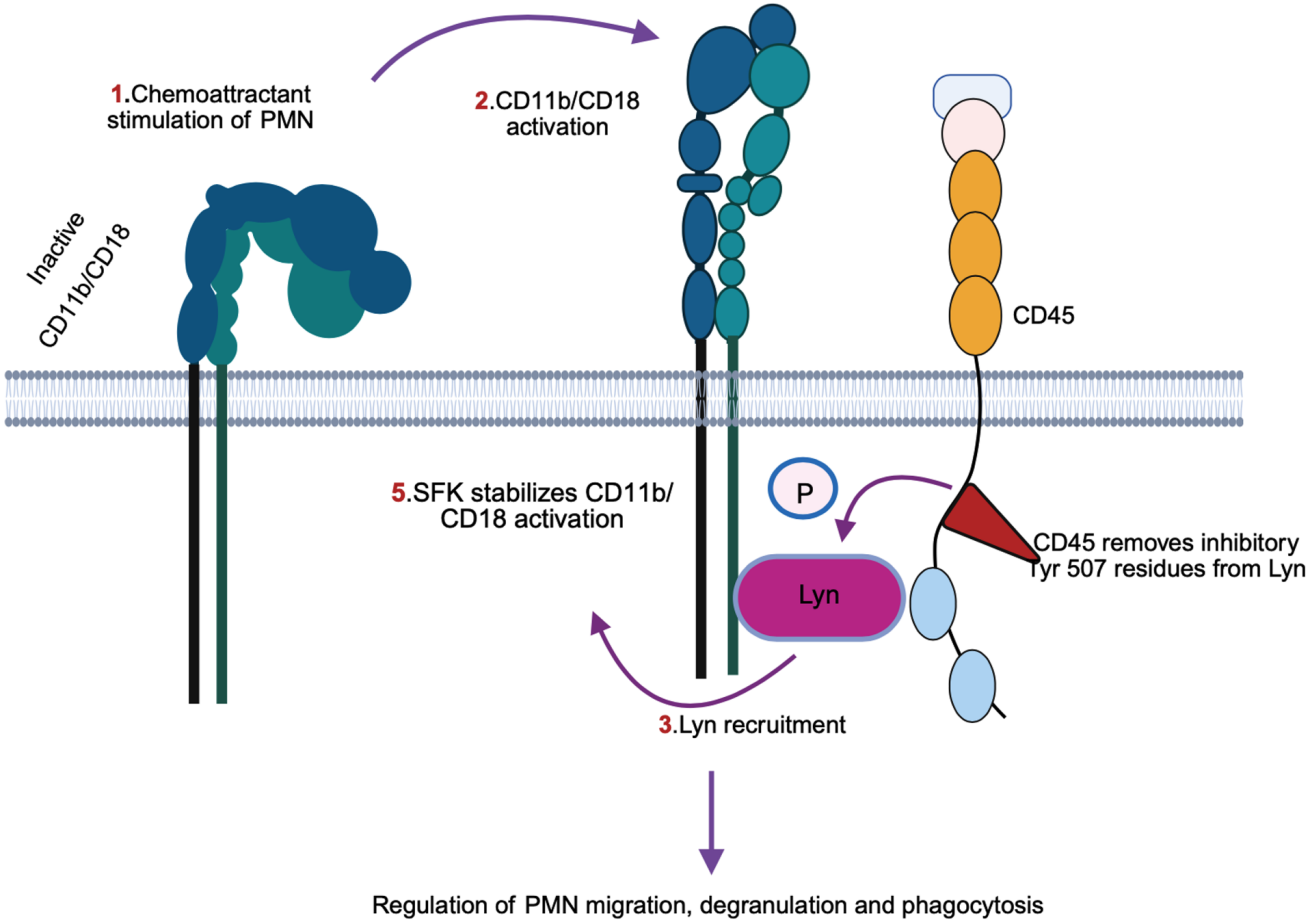
Model figure showing how CD11b recruits Lyn kinase, which is activated by CD45 phosphatase, in turn stabilizing CD11b/CD18 surface expression and activation and promoting PMN antimicrobial effector functions.

Given previous studies showing integrin-mediated recruitment of SFKs (23–25) in cancer cells, our data support the model shown in Figure 12, whereby upon PMN stimulation active CD11b/CD18 brings Lyn kinase into close physical association with CD45, resulting in removal of the inhibitory phosphorylation motif at Tyr507, thus stabilizing CD11b/CD18 surface expression/activation and driving PMN effector functions necessary for pathogen clearance and mucosal wound repair.

## Discussion

While dysregulated or excessive PMN trafficking to mucosal tissues is implicated in the pathogenesis of numerous inflammatory disorders (26–28), insufficient PMN tissue trafficking is linked to chronic infections and delayed mucosal repair (29–31). Therefore, it is vitally important to uncover molecular mechanisms that can be exploited to maximize beneficial PMN effector functions, including pathogen clearance and tissue repair while minimizing potentially damaging prolonged PMN responses. Several studies have shown that expression and glycosylation of the β2 integrin CD11b/CD18 regulates PMN intracellular signaling, PMN trafficking, and PMN effector functions (9–11, 20). However, the complex intracellular signaling cascades that regulate PMN function in mucosal tissues remain incompletely understood. While the PTP CD45 has been described as a critical regulator of adaptive immune cell function (7, 8, 32–34), much less is known about how CD45 regulates PMN function during mucosal inflammation and repair.

Here we demonstrate that chemical inhibition of CD45 phosphatase activity with CD45 inhibitor VI potently restricts PMN trafficking into the intestine in vitro and in vivo. Importantly, it has been demonstrated that while CD45 inhibitor VI exhibits robust inhibition of CD45, it has minimal inhibitory effects (IC_50_ > 40 μM) on several related PTPs, including MKPX, PRL-2, PTP1B/PTPN1, TC-PTP/PTPN2, SHP-1/PTPN6, PEP/PTPN22, LAR/PTPRF, and PTP-sigma/PTPRS (12). Observed reductions in PMN transmigration responses are in keeping with previous work showing that a specific mAb against CD45 decreased PMN chemotaxis in vitro (35). In support of this, here we show that exposure of human PMNs to CD45 inhibitor VI results in reduced chemotaxis across collagen-coated Transwell filters, suggesting that CD45-mediated signaling plays a role in modulating multiple types of PMN trafficking. In keeping with this, here we show inhibition of human PMN TEpM by the anti-CD45 mAb MEM-28, further highlighting that engagement of the extracellular domain (ECD) of CD45 regulates phosphatase activity and downstream signaling in PMNs. CD45 contains a highly glycosylated ECD that can include up to 3 alternatively spliced exons (exons A, B, and C), depending on cell type and activation status (36–38). Furthermore, it has been demonstrated in T cells that CD45 ECD dimerization repositions the intracellular domains in a way that reduces access to substrates and lowers CD45 phosphatase–mediated signaling (39). Previous studies on B and T cells have also demonstrated that CD45 phosphatase activity can be regulated by binding interactions between lectins, including Galectin-1, Galectin-3, and CD22 and glycan structures on the CD45 ECD (40–42). However, future studies are needed to determine the importance of CD45 ligand binding in PMN cell signaling and function during mucosal inflammation and repair.

Decreased PMN intestinal trafficking was observed in *MRP8-Cre;Cd45^fl/fl^* mice in the current study. However, in contrast, no difference in PMN recruitment to the lung was observed in total CD45KO mice infected with *Staphylococcus* *aureus* (43). Such discrepancies could be explained by the fact that dysregulated T and B cell responses and associated immunodeficiencies (18) complicate interpretation of PMN function in global CD45KO mice. Alternatively, differences in regulation of PMN migration mediated by CD45 could be organ specific. In support of CD45 regulating PMN intestinal trafficking during an inflammatory response, here we show decreased PMN intestinal trafficking in PMN-specific CD45-deficient mice subjected to DSS-induced colitis.

In addition to regulated trafficking, tight control of other key PMN effector functions is essential for effective host immune defense and tissue repair. Here we report reduced PMN degranulation and phagocytosis responses upon CD45 inhibition or knockdown. These data suggest that CD45-deficient PMNs in mucosal tissues have a reduced capacity to engulf and eliminate pathogens. In keeping with findings of reduced phagocytosis upon CD45 depletion or inhibition, a previous study revealed that PMNs from total CD45KO mice display a subtle (although not statistically significant) reduction in clearance of *S*. *aureus* in a lung pouch inflammation model (43). However, in contrast to such subtle reductions in murine PMN phagocytosis, here we report robust and significant reductions in phagocytosis in WT murine PMNs or human PMNs exposed to CD45 inhibitor VI, in CD45KO HL60 cells and in PMNs from *MRP8-Cre;Cd45^fl/fl^* mice. In further support of CD45-mediated regulation of PMN phagocytosis, it has been previously shown that antibody-mediated crosslinking/activation of CD45 increased phagocytosis of fluorescent beads by human PMNs (44). However, in that study, effects of CD45 crosslinking on PMN phagocytosis were erroneously attributed to changes in phosphorylation of Lck, a lymphocyte-specific SFK since described to not be expressed in PMNs (45, 46).

Functionally, we show that mice with PMN-specific deletion of CD45 exhibit delayed recovery from DSS colitis and have increased levels of ulceration, inflammation, weight loss, and occult bleeding. We also report decreased PMN trafficking through inflamed intestinal lamina propria in the absence of CD45 expression. Mice with CD45-deficient PMNs also exhibited reduced colonic wound repair following biopsy-induced colonic injury. These data represent the first report to our knowledge showing functional effects on intestinal inflammation and repair in the absence of PMN CD45 phosphatase activity. Given that CD45 inhibition or depletion was shown to decrease intestinal trafficking as well as phagocytosis and degranulation, it is possible that delayed repair observed in *MRP8-Cre;Cd45^fl/fl^* mice is due to insufficient trafficking of phagocytosis/degranulation-deficient PMNs to the intestine, leading to decreased pathogen clearance and prolonged tissue inflammation.

While many PMN functions, including TEpM, have been shown to be regulated by glycosylation and activation of the β2 integrin CD11b/CD18 (9–11), studies under conditions of CD45KO where PMN CD11b/CD18 levels or activation states are measured have not been previously performed. Therefore, we explored changes in CD11b surface expression and activation in the absence of PMN CD45 phosphatase activity or in the absence of CD45 expression. Interestingly, we report reduced surface expression of CD11b/CD18 in murine and human PMNs exposed to CD45 inhibitor VI, in CD45KO HL60 cells, and in PMNs from *MRP8-Cre;Cd45^fl/fl^* mice. Furthermore, using a reporter antibody specific for activated human CD11b/CD18, we observed decreased levels of CD11b activation in CD45KO HL60 cells and human PMNs exposed to CD45 inhibitor VI. These data suggest that potent functional effects observed upon CD45 inhibition or deletion are in part a consequence of decreased PMN CD11b/CD18 surface expression/activation, highlighting important crosstalk between CD11b/CD18 and CD45. In support of this, it has been demonstrated that PMNs from CD45E613R mice (which have a single point mutation that results in constitutive activation of CD45) exhibit increased CD11b/CD18-mediated adhesion to vascular endothelial cells (47).

Previous studies have demonstrated that CD11b/CD18 activity is in part modulated by inside-out signaling, which can be regulated by tyrosine phosphorylation of SFKs. PMNs are reported to express 3 SFK members: Hck, Fgr, and Lyn (43). In support of a connection between SFK signaling and integrin function, PMNs from *Hck*^–/–^, *Fgr*^–/–^, and *Lyn*^–/–^ triple KO mice display impaired CD11b/CD18-mediated adhesion to platelets (48). CD45 has previously been identified as a critical PTP that regulates specific SFK members, including Lck, which is expressed in T and B cells but not in PMNs (43). Here we show specific CD45-mediated dephosphorylation of Lyn kinase but not the other PMN-expressed SFK members Hck and Fyn. The C-terminus of Lyn kinase compromises the protein tyrosine kinase (PTK) domain and a short tail containing a regulatory residue (Tyr507) that when phosphorylated switches Lyn to an inactive closed conformation with limited substrate access to the PTK domain (49). Importantly, here we show that when CD45 is inhibited or depleted, Tyr507 dephosphorylation does not occur, suggesting persistent PMN Lyn deactivation in the absence of CD45 phosphatase activity. Furthermore, given that Lyn is not expressed in T cells, being downregulated at the thymocyte double-negative stage of development (49), data suggest that while CD45 expression is somewhat ubiquitous in hematopoietic cells, it can differentially regulate intracellular signaling transduction in a cell- and kinase-specific fashion, with Lck being important for T cells (50, 51) and data shown here identifying Lyn as being an important CD45 target in PMNs. Further highlighting the biological significance of the signaling between CD45, Lyn, and β2 integrins, PMNs from patients with small vessel vasculitis caused by gain-of-function mutations in Lyn kinase exhibited increased surface expression of CD11b/CD18 as detected by flow cytometry (52). While previous studies have shown activation of the SFKs Lyn and Fgr upon cross-linking of PMN surface integrins (53) and, that PMNs lacking all 3 myeloid SFKs (Hck, Fgr, and Lyn) are unable to spread on integrin-specific antibody–coated surfaces (54), data shown here represent the first report to our knowledge of a specific CD45/CD11b/Lyn kinase signaling axis regulating PMN trafficking and effector function in the intestine. In support of the importance of Lyn kinase for regulating PMN function downstream of CD45, here we show that inhibition of Lyn with Dasatinib recapitulates functional effects of CD45 inhibition or deletion in PMNs. Previous studies have reported that Dasatinib can inhibit multiple SFK members in addition to Lyn kinase (55, 56). However, signaling data revealed no change in phosphorylation of the other PMN-expressed SFK members (Hck and Fgr) in differentiated HL60 cells exposed to Dasatinib, suggesting that functional effects of Dasatinib on PMN function are being mediated specifically via Lyn kinase inhibition. Additionally, inhibition of PMN degranulation and phagocytosis were also observed using a second Lyn kinase inhibitor, Bafetinib. Importantly Bafetinib has only 2 known targets (Lyn and BCR-Abl) (57), with BCR-Abl reported to not be expressed in mature PMNs under normal circumstances (58). Taken together, these data provide strong evidence that in activated PMNs CD45 signals through Lyn kinase and CD11b to regulate critical PMN functional responses, including migration, degranulation, and phagocytosis.

## Methods

### Sex as a biological variable.

For human PMN isolation, an equal number of male and female blood donors were used for studies and no difference in PMN response was observed between sexes. Similarly, for all mouse experiments our study examined male and female animals and similar findings are reported for both sexes.

### Antibodies and other reagents.

Mouse anti-human CD45 mAb (MEM-28, catalog ab8216) was purchased from Abcam. Rabbit anti-human CD45 mAb for Western blotting was purchased from Proteintech (catalog 20103-1-AP). APC- or PercP-conjugated rat anti-mouse CD45 mAb (clone 30-F11, catalog 103112), APC-conjugated IgG control mAb (clone RTK4530, catalog 103112), Brilliant Violet 605–conjugated rat anti-mouse Ly6G (clone 1A8), Alexa Fluor 647–conjugated rat anti-mouse F4/80 (clone BM8), phycoerythrin-conjugated (PE-conjugated) rat anti-mouse CD11b (clone M1/70), PE-Cy7–conjugated anti-Ly6c (clone HK1.4), BV421-conjugated anti–Siglec-F (clone E50-2240), BV605-conjugated anti-CD4 (clone GK1.5), PE-conjugated anti-CD3 (clone 145-2C11), and PerCP-conjugated anti-CD8 (clone 53-6.7) were purchased from BioLegend. eFluor 450–conjugated rat anti-mouse Ly6C (clone HK1.4), eFluor 450–gdTRC (clone ebioGL3), PE-Cy–conjugated anti-CD19 (clone eBioID3), and eFluor 780 live/dead stain were purchased from eBioscience.

Polymorphprep was purchased from Axis-Shield. FITC- or PE-conjugated FluoSpheres (1 mm) were purchased from Molecular Probes. fMLF, LaB, CD45 inhibitor VI, Dasatinib, and anti–p-Hck Tyr521 mAb (catalog SAB4301379) were purchased from Sigma-Aldrich. FITC- or PE-conjugated CD11b activation specific monoclonal antibody (CBRM1/5) and enzyme-free cell dissociation buffer were purchased from Thermo Fisher Scientific (catalog 14-0113-81). Bafetinib was purchased from Cayman Chemical. Antibodies for total Hck (catalog 14643), total Fgr (catalog 2755), p-Fgr Tyr412 (catalog 48984), total Lyn (catalog 2732), and Lyn Tyr507 (catalog 2731) were purchased from Cell Signaling Technologies. Human TruStain FcX (Fc Receptor Blocking Solution), FITC-conjugated mouse anti-human CD11b mAb (clone ICRF44), and APC-conjugated rat anti-mouse CD11b mAb (clone M1/70) were purchased from BioLegend. Mouse anti-human FITC-conjugated anti-CD63 and anti-CD66b mAbs, PERCP-conjugated rat anti-mouse CD45 (clone 30-F11), and APC-Cy7–conjugated anti-mouse Siglec-F (clone E50-2440) were purchased from BD Biosciences. Rat anti-mouse PE-conjugated CD63 (catalog 12063182) and anti-CD15 (catalog MA1-022) mAbs were purchased from Thermo Fisher Scientific.

### Cell culture and PMN isolation.

T84 IECs were cultured as described previously (9, 10, 13). Human PMNs were isolated from whole blood obtained from healthy male and female donors, with approval from the University of Michigan Institutional Review Board (IRB) on human subjects, using a previously described polymorph density gradient centrifugation technique (13, 14, 59, 60). Isolated PMNs were 98% pure and greater than 95% viable and were used for all described assays within 2 hours of blood draw. Murine neutrophils were isolated from bone marrow extracted from the femurs and tibias of male and female C57BL/6 mice using EasySep kits from Stem Cell Technologies, as previously described (11). Human myelomonocytic HL60 cells were obtained from ATCC (clone CCL-240). For all assays, HL60 cells were cultured in RMPI-1640 supplemented with 20% FBS, 100 U/mL penicillin, 100 μg/mL streptomycin, 2 mM L-glutamine, and 1% nonessential amino acids at 37°C in a 5% CO_2_ incubator and differentiated into a PMN-like phenotype using a slightly modified published technique (17). Specifically, HL60 cells were differentiated into a PMN-like phenotype by supplementing growth media with 250 nM all-*trans* retinoic acid for 1 day followed by addition of 1.25% DMSO for 4 days.

### Plasmid construction for CD45KO HL60 cells.

The SpCas9 of lentiCRISPR V2 (Addgene plasmid 52961) was modified to generate a plasmid containing the high-fidelity eSpCas9 1.1 (61) termed lentiCRISPR eSpCAS9. CRISPOR (62) was used to identify potential guides to target human CD45 exon 2. CD45 targeting and a scrambled sequence were cloned into the BsmBI site of lentiCRISPR eSpCAS9 to generate CD45-targeting plasmids and a SCR control. The target sequences are as follows: CD45KO: 5′-GAGTTTAAGCCACAAATACA, SCR: 5′-GCACTACCAGAGCTAACTCA. All clones were sequence verified, and plasmid DNA prepared using standard methodology. Individual lentiviruses were produced by the University of Michigan vector core using standard lentivirus production methodology. Transduced CD45KO HL60 cells were cultured for 5 to 7 days in RPMI 1640 supplemented with 10% fetal calf serum and L-glutamine before depletion of CD45-positive cells using an anti-CD45 mAb (MEM-28) and magnetic beads coated with sheep anti-mouse IgG Dynabeads (Life Technologies).

### PMN TEpM assay.

T84 IECs were grown on collagen-coated, permeable 0.33 cm^2^ polycarbonate filters (3 mm pore size) as inverted monolayers. Effects of 500 nM CD45 inhibitor VI or 10 mg/mL anti-CD45 mAb MEM-28 (along with appropriate controls) on human PMN TEpM to 100 nM fMLF in the physiologically relevant basolateral to apical direction assessed by myeloperoxidase quantification, as described previously (11, 13, 59, 60). For migration across primary IECs, human colon sample collection was performed in accordance with the University of Michigan IRB regulations. Isolated colonoids were resuspended in Matrigel and cultured in growth medium (50% L-WRN conditioned medium: 50% advanced DMEM/F-12, 10% FBS, 2 mM GlutaMax, 10 mM HEPES, N-2 media supplement, B-27 supplement, 1 mM *N*-acetyl-L-cysteine, 50 ng/mL human EGF, 100 U/mL penicillin, 0.1 mg/mL streptomycin, 500 nM A83-01, 10 μM SB202190, 10 mM nicotinamide, 10 nM gastrin). To generate 2D monolayers, colonoids grown as described above were spun out of Matrigel and dissociated into a single-cell suspension according to published protocols (15, 63) and plated as inverted monolayers on collagen-coated, permeable 0.33 cm^2^ polycarbonate filters (3 mm pore size). Human colonoids formed confluent polarized inverted monolayers with TEER values greater than 1,000 Ω•cm^2^ within 5–7 days. Effects of 500 nM CD45 inhibitor VI or 10 mg/mL anti CD45 mAb MEM-28 (along with appropriate controls) on human PMN TEpM to 100 nM fMLF in the physiologically relevant basolateral to apical direction assessed by myeloperoxidase quantification as described above.

### Generation of PMN-specific CD45 knockdown mice.

All mice used in this study were on the C57BL/6 background and were maintained in breeding colonies established in the specific pathogen–free facility at the University of Michigan School of Medicine. Animals were maintained under specific pathogen–free conditions with a 12-hour day/night cycle and access to food and water ad libitum. For CD45 inhibitor studies, male and female C57BL/6 WT mice aged between 10 and 14 weeks were used. To generate PMN-specific CD45 knockdown mice, heterozygous *Cd45^WT/fl^* mice (C57BL/6NCya-*Ptprcem1^fl^*/Cya) purchased from Cyagen were crossed to produce homozygous *Cd45^fl/fl^* offspring. Subsequently, *Cd45^fl/fl^* mice were crossed with mice constitutively expressing Cre under control of the granulocyte promoter *MRP8-Cre-IRES/GFP* purchased from The Jackson Laboratory to generate *MRP8-Cre;Cd45^fl/fl^* mice with targeted deletion of *Cd45* in PMNs.

### Colonic loop model of in vivo PMN TEpM.

Colon loop experiments were performed with male and female C57BL/6 mice aged 8–12 weeks, which were maintained under standard conditions with 12-hour light/12-hour dark cycles and ad libitum access to food and water. PMN TEpM into the proximal colon was assessed using a previously described in vivo surgical model (15, 16). Briefly, mice were pretreated with proinflammatory cytokines before a 2 cm loop of fully vascularized proximal colon was injected with 1 nM LTB_4_ in conjunction with intraluminal injection of 500 nM CD45 inhibitor VI or intraperitoneal injection of 3 mg/kg body weight CD45 inhibitor VI along with relevant concentrations of vehicle control. Quantification of absolute numbers of PMNs (CD45^+^CD11b^+^LyG^+^) migrated into the colonic lumen was performed by flow cytometry.

### Flow cytometric assessment of CD11b surface expression and activation.

For all flow cytometry experiments, cells were blocked with 3% bovine serum albumin containing Human or Murine TruStain FcX (Fc Receptor Blocking Solution, BioLegend). For assessment of surface expression of CD11b/CD18, human PMNs or differentiated HL60 cells were incubated with a FITC-conjugated anti-CD11b mAb (clone ICRF44). For assessment of CD11b/CD18 activation, human PMNs or differentiated HL60 cells were incubated with FITC-conjugated CBRM1/5. For assessment of CD11b/CD18 surface expression in murine PMNs, cells were incubated with an APC-conjugated anti-CD11b/CD18 mAb (clone M1/70). Flow cytometric analyses were performed using a NovoCyte Flow cytometer (ACEA Bioscience).

### Transcriptional analysis.

Murine PMNs isolated as described above or HL60 cells were lysed in TRIzol (Thermo Fisher Scientific) and then subjected to phenol-chloroform extraction, according to the manufacturer’s protocol. RNA was digested with DNase I (Ambion) to remove contamination with genomic DNA; then, DNA was synthesized by reverse transcription using oligo(dt12–18) primers and Superscript II reverse transcriptase (Thermo Fisher Scientific). Real-time PCR was performed with a MyIQ real-time PCR machine and SYBR Green supermix (Bio-Rad Laboratories) using primers for murine *Cd45* (Origene, MP222432) and human *CD45* (Origene, HP206460). Data were analyzed by the ΔΔC_t_ threshold cycle method and normalized to the housekeeping gene *GAPDH*. Data are reported as mean ± SEM from 3 *MRP8-Cre;Cd45^fl/fl^* mice and 3 *Cd45^fl/fl^* mice or from 3 passages of HL60 cells.

### PMN degranulation and phagocytosis assays.

For degranulation assays, murine PMNs, human PMNs, or HL60 cells differentiated to a PMN-like state were incubated with 500 nM CD45 inhibitor VI for 30 minutes at 37°C. As a positive control for degranulation, cells were exposed to 1.25 mM LaB for 5 minutes followed by stimulation with either 5 mM fMLF (human PMNs) or 10 mM fMLF (mouse PMNs and HL60 cells). For analysis of degranulation of adherent cells, differentiated HL60 cells or human PMNs adhering to 5 mg/mL fibronectin were exposed to 1.25 mM LaB for 5 minutes followed by stimulation with either 5 mM fMLF (human PMNs) or 10 mM fMLF (HL60 cells). For degranulation of adherent PMNs without priming by LaB, human PMNs adhering to fibronectin were stimulated with 100 nM PMA. After indicated stimulations, cells were detached using enzyme-free cell dissociation buffer before washing and incubation with human TruStain FcX (Fc Receptor Blocking Solution) and incubation with FITC- or PE-conjugated mAbs against CD63 or CD66b and data acquisition using a NovoCyte flow cytometer (ACEA Bioscience). For phagocytosis assays in suspension, PMNs were incubated with 100 nM Bafetinib, 250 nM Dasatinib, or vehicle control before incubation with FITC- or PE-conjugated FluoSpheres at a ratio of 1:100 (PMNs/FluoSpheres) in the presence of 10 nM fMLF. Uptake of FluoSpheres by PMNs was assessed by measurement of intracellular fluorescence by flow cytometry. For phagocytosis of pHrodo-conjugated *E*. *coli*, murine PMNs or PMN-like HL60 cells (either in suspension or adhering to 5 mg/mL fibronectin) were incubated with 100 nM Bafetinib, 250 nM Dasatinib, or vehicle control along with 20 mg pHrodo-conjugated *E*. *coli* bioparticles followed by incubation for 1 hour at 37°C in the presence of 10 nM fMLF before cell assessment of intracellular fluorescence by flow cytometry as described above. As a control, levels of phagocytosis at 37°C were compared to levels of phagocytosis at 4°C.

### DSS-induced colitis and histological scoring.

For DSS colitis and recovery experiments, *MRP8-Cre;Cd45^fl/fl^* mice or control *Cd45^fl/fl^* mice were administrated 3% DSS via drinking water for 5 days followed by 5 days of regular water recovery. A composite Disease Activity Index (DAI) score was calculated daily each day by measuring body weight, stool consistency, and the presence of occult blood as described previously (15, 64).

For histological scoring, 6-μm H&E-stained Swiss roll sections were analyzed by 2 independent investigators using an Aperio ImageScope 12 (Leica Biosystems). Histological scoring of H&E-stained tissue sections of colonic mucosa was performed where histological colitis score represents a ratio of the length of injured/ulcerated areas relative to the entire colon length, as assessed in Swiss roll mounts of the entire colon. Subsequently, percentages of inflamed and ulcerated areas were calculated and a final score generated using a validated scoring system previously published by our group (65).

### In vivo biopsy wounding of colonic mucosa.

Biopsy wounding of colonic mucosa in mice was performed as previously described (15). Briefly, mice were anesthetized by an intraperitoneal injection of a ketamine (100 mg/kg)/xylazine (10 mg/kg) solution. Biopsy-induced injuries of the colonic mucosa were made along the mesenteric artery using a high-resolution, miniaturized colonoscope system equipped with a biopsy forceps (Colorview Veterinary Endoscope, Karl Stortz). Six to 10 lesions were generated per animal. Endoscopic procedures were viewed on day 1 (24 hours) and day 3 (72 hours) after injury with high-resolution images (1,024 × 768 pixels) on a flat-panel monitor.

### Flow cytometric analysis of bone marrow, circulating, or colonic lamina propria immune cells.

Whole blood was removed from anesthetized mice by cardiac puncture and red blood cells lysed using ACK buffer. Immune cells were stained with a labeled primary antibody cocktail in the presence of Fc block for 30 minutes at 4°C and analyzed by flow cytometry. For bone marrow analysis, immune cells were flushed out of the femurs and tibias of euthanized mice before red blood cell lysis with ACK buffer. Isolated immune cells were stained with a labeled primary antibody cocktail in the presence of Fc block for 30 minutes at 4°C and analyzed by flow cytometry. For isolation of lamina propria cells, mouse Lamina Propria Dissociation Kits (Miltenyi Biotec) were used according to the manufacturer’s protocols. Isolated lamina propria cells were resuspended in flow buffer (PBS containing 2% FBS/1 mM EDTA) and filtered through a 70-μm nylon mesh. Cells were stained with a labeled primary antibody cocktail in the presence of Fc block for 30 minutes at 4°C and analyzed by flow cytometry.

### Cell lysis and immunoblotting.

For cell signaling analysis or detection of CD45 expression by Western blotting, PMNs or differentiated HL60 cells were directly lysed in boiling 2× Laemmli buffer and immunoblotting performed as described previously (13, 15).

### Statistics.

Statistical analyses were performed using Prism software (GraphPad Software Inc.). Statistical differences were evaluated by unpaired, 2-tailed Student’s *t* test or 1-way ANOVA followed by Tukey’s multiple-comparison test. A *P* value of less than 0.05 was considered statistically significant. Data are presented as mean ± SEM, whereby each data point represents 1 individual biological replicate. All results show data from at least 2 independent experiments.

### Study approvals.

All experimental procedures involving animals were conducted in accordance with the NIH *Guide for the Care and Use of Laboratory Animals* (National Academies Press, 2011) and protocols approved by the University Committee on Use and Care of Animals at the University of Michigan.

### Data availability.

Supporting data for all figures are available from the corresponding author upon request. Values for all data points in graphs are reported in the Supporting Data Values file.

## Author contributions

JCB designed the study, performed data collection, and performed data analysis/interpretation. JM, DJF, RH, and ZSW performed experiments for the study. JCB wrote the manuscript. AN and CAP provided assistance in writing the manuscript.

## Conflict of interest

The authors have declared that no conflict of interest exists.

## Funding support

This work is the result of NIH funding, in whole or in part, and is subject to the NIH Public Access Policy. Through acceptance of this federal funding, the NIH has been given a right to make the work publicly available in PubMed Central.

- NIH grants R56DK140172 and R01DK140172 (to JCB), R01DK055679 and R01DK059888 (to AN), and R01DK129058, R01DK129214, and R01DK079392 (to CAP).

## Supplementary Material

Supplemental data

Unedited blot and gel images

Supporting data values

## Version 1 06/23/2026

In-Press Preview

## Version 2 08/10/2026

Electronic publication
