# Supplementary material for: The protein tyrosine phosphatase CD45 promotes PMN transepithelial migration, antimicrobial function, and colonic mucosal repair
Source: JCI Insight. 2026 Jun 23;11(15):e203438. doi: 10.1172/jci.insight.203438 (PMC13463622; doi:10.1172/jci.insight.203438)
Supplement: Supplementary file 1 [file jciinsight-11-203438-s072.pdf]

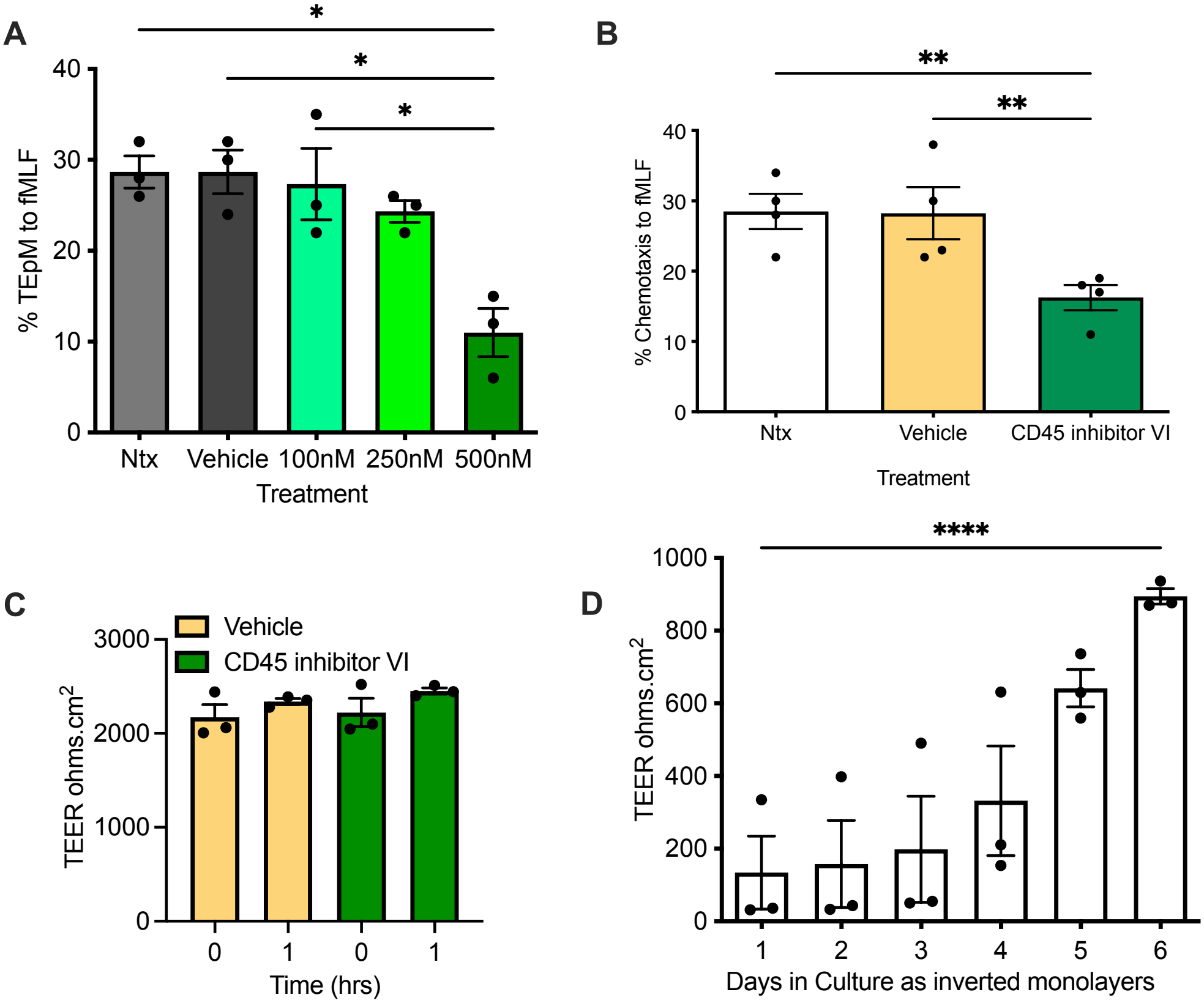

Supplemental Figure 1. Dose response of CD45 inhibitor VI on PMN TEpM, effect of CD45 inhibitor VI on PMN chemotaxis, effect of CD45 inhibitor VI on T84 IEC barrier development and development of barrier in primary 2D colonoids. (A) 1 x 10^6 human PMNs were incubated with 100-500nM CD45 inhibitor VI or vehicle control before being added to the basolateral surface of confluent inverted T84 monolayers. PMNs were allowed to migrate in the physiologically relevant basolateral to apical direction for 1 hour in response to a 100nM gradient of n-formyl-methionyl-leucyl-phenylalanine (fMLF). The number of migrated PMNs were quantified by myeloperoxidase assay. Data are means ± SEM (n=3 donors per group, * $p<0.05$). (B) 1 x 10^6 human PMNs were incubated with 500nM CD45 inhibitor VI or vehicle control before being added to the basolateral surface of collagen coated transwells. before being allowed to migrate for 1 hour in response to a 100nM gradient of n-formyl-methionyl-leucyl-phenylalanine (fMLF). The number of migrated PMNs were quantified by myeloperoxidase assay. Data are means ± SEM (n=4 donors per group, ** $p<0.01$). (C) TEER was measured in confluent inverted T84 monolayers before and after addition of 500nM CD45 inhibitor VI or vehicle control for 1 hour. Data are means ± SEM for 3 independent experiments with 6 transwells per condition in each experiment. (D) Human colonoids were seeded as inverted 2D monolayers and TEER measured daily using an EVOM. Data shown are means for 3 independent experiments with 3 replicate transwells per condition in each experiment, **** $p<0.0001$. Data represent means ± SEM and were analyzed by 1-way ANOVA followed by Tukey's post hoc testing.

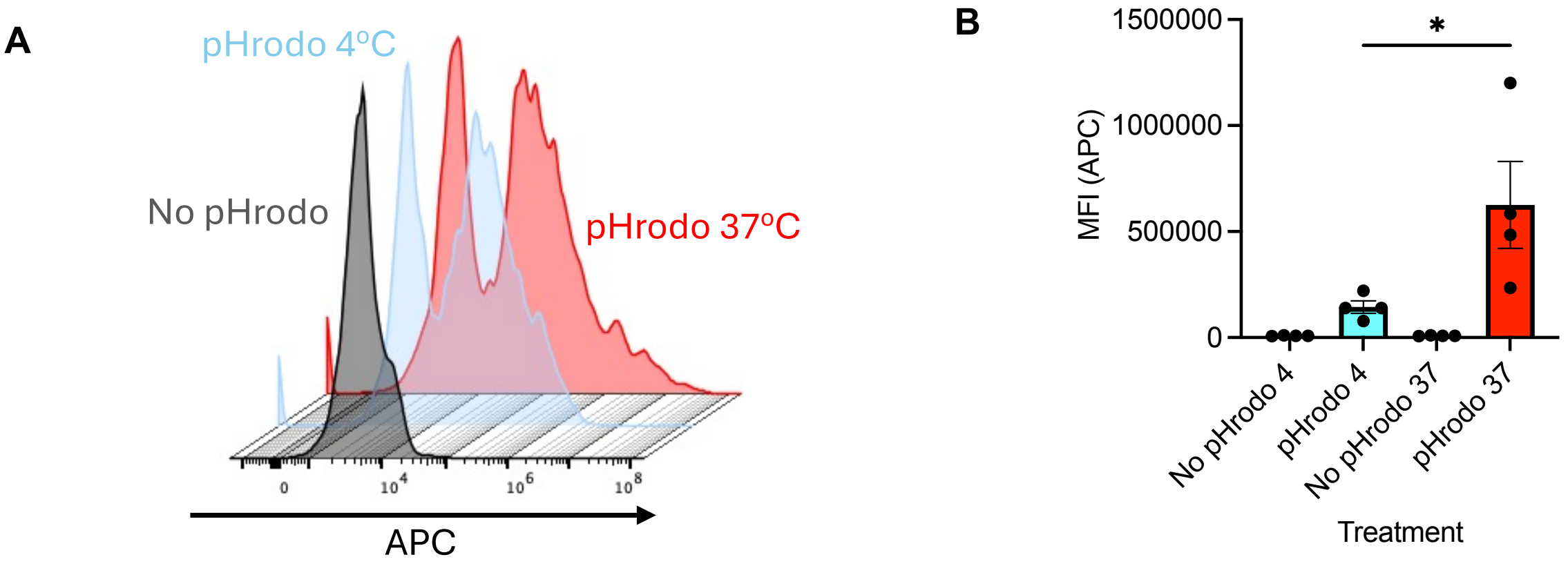

Supplemental Figure 2. Reduced phagocytosis of *E.coli* bioparticles observed at 4°C. Differentiated HL60 cells were incubated with 20µg pHrodo conjugated *E.coli* bioparticles in the presence of 10nM fMLF at either 4°C or 37°C. Phagocytosis was quantified by measuring changes in APC fluorescence by flow cytometry. Data are mean fluorescence intensity for APC presented as means ± SEM and were analyzed by 1-way ANOVA followed by Tukey's post hoc testing (n=3 independent biological experiments,* $p<0.05$).

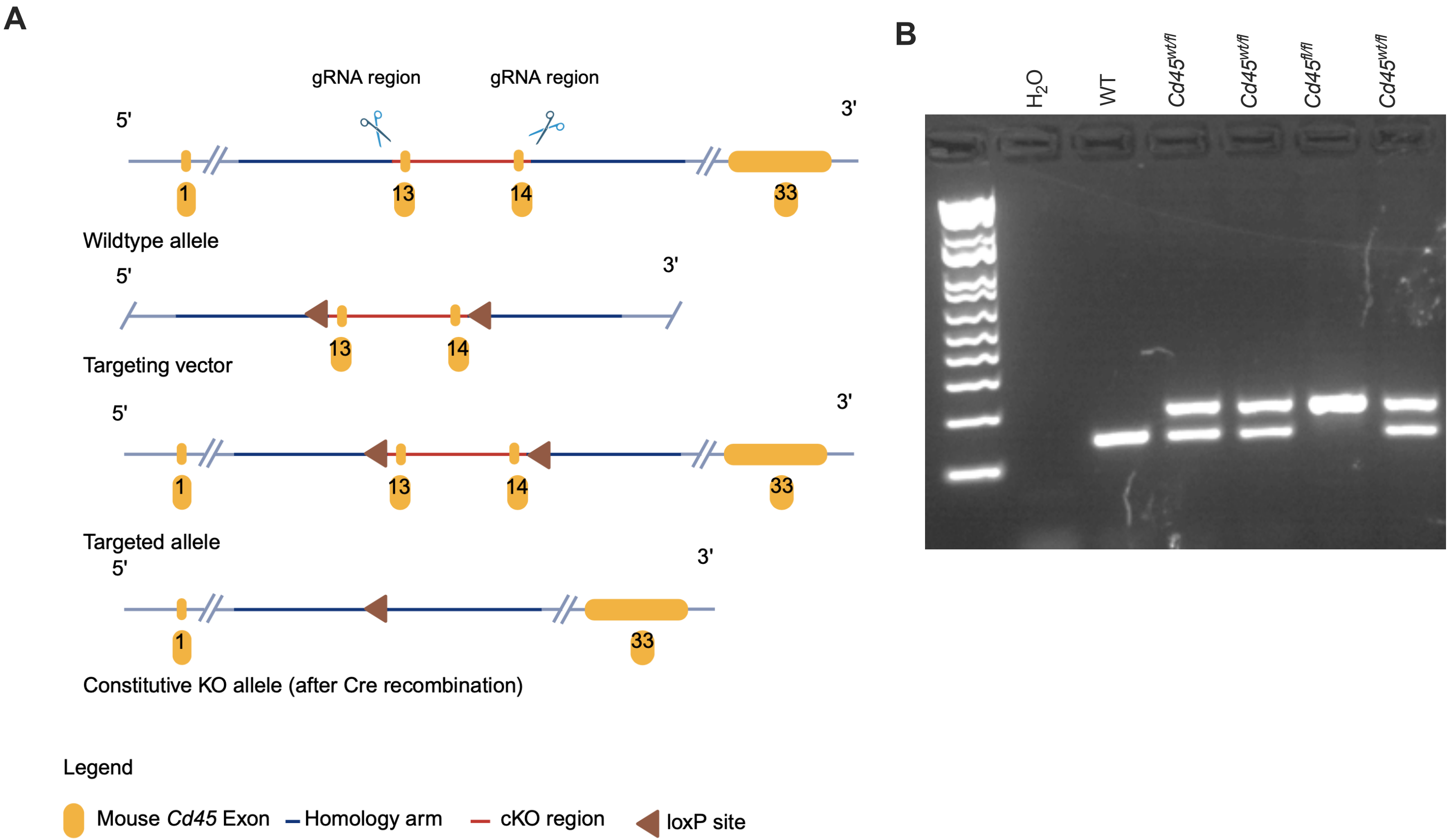

Supplemental Figure 3. Generation of homozygous *Cd45*$^{fl/fl}$ mice through crossing of *Cd45*$^{wt/fl}$ heterozygotes. (A) Cartoon showing excision of the *Cd45* exon by Cre recombinase. (B) PCR genotyping showing a wildtype (WT) mouse (single lower band), three Cd45$^{wt/fl}$ heterozygotes (two bands) and a *Cd45*$^{fl/fl}$ homozygote (single upper band)

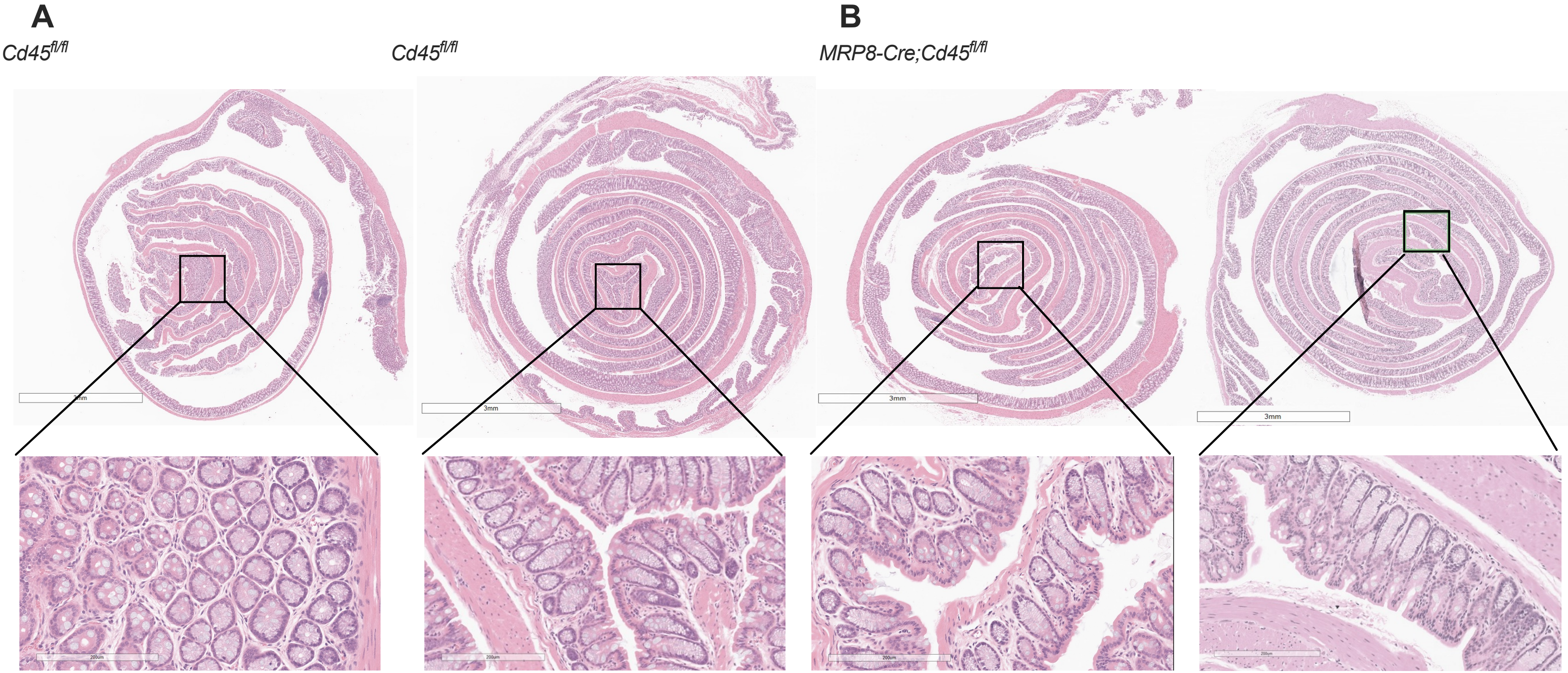

Supplemental Figure 4. Mice with PMN specific CD45 depletion have normal intestinal architecture. Hematoxylin and Eosin staining of intestinal Swiss roles from *Cd45*$^{fl/fl}$ mice (A) and *MRP8-Cre;Cd45*$^{fl/fl}$ mice (B) showing normal intestinal architecture.

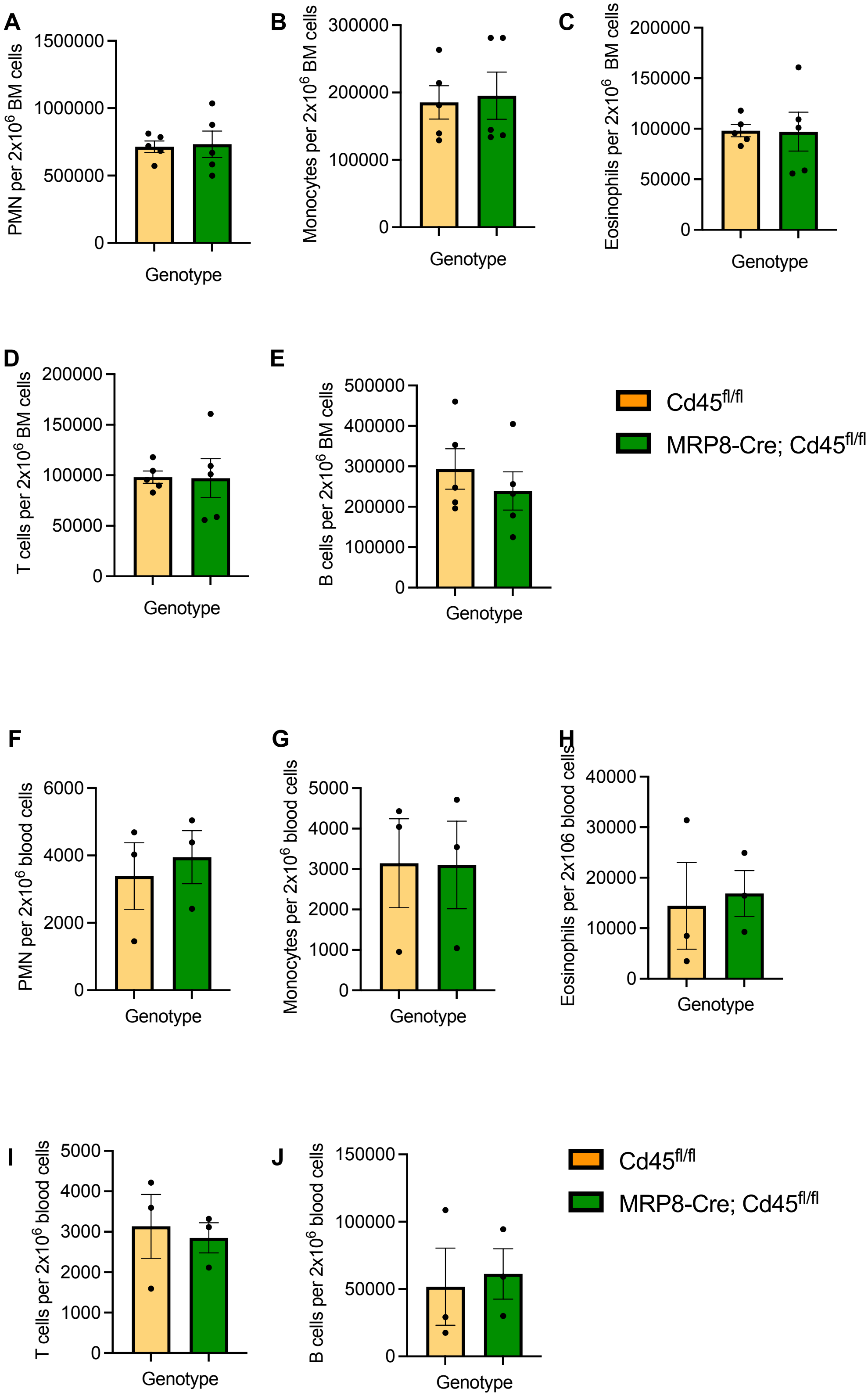

Supplemental Figure 5. Mice with CD45 deficient PMN have normal numbers of bone marrow immune cells and circulating immune cells. Immune cell numbers per 2 x 10^6 bone marrow derived cells isolated from *Cd45$^{fl/fl}$* mice and *MRP8-Cre;Cd45$^{fl/fl}$* mice. Quantification of flow cytometry data showing numbers of bone marrow (A-E) or blood derived (F-J) PMN (SiglecF^{+ve}, CD11b^{+ve},LygG^{+ve}, Ly6C^{+ve}), Monocytes (SiglecF^{+ve}, CD11b^{+ve},LygG^{+ve}, Ly6C^{+ve}), Eosinophils (SiglecF^{+ve}, CD11b^{+ve}), B cells (CD19^{+ve}, CD3^{-ve}) and T cells (CD4^{+ve}, CD3^{+ve}) from *MRP8-Cre;Cd45$^{fl/fl}$* mice compared to cells from *Cd45$^{fl/fl}$* control mice. Data represent means ± SEM and were analyzed by unpaired two tailed t test (n=3-5).

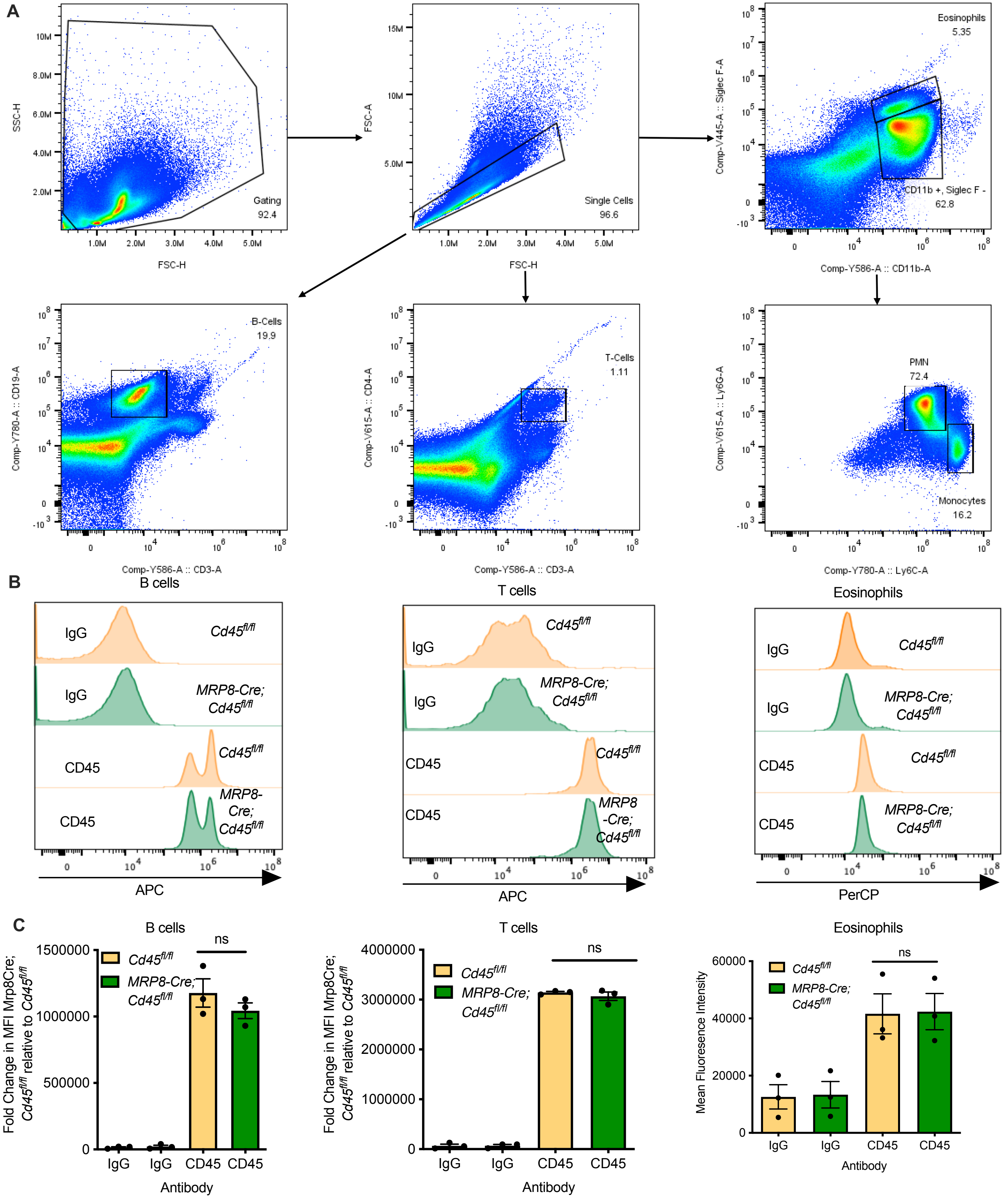

Supplemental Figure 6. CD45 Surface Expression on bone marrow derived immune cells isolated from *Cd45*$^{fl/fl}$ mice and *MRP8-Cre;Cd45*$^{fl/fl}$ mice. (A) Gating strategy. (B) Representative flow cytometry plots of bone marrow derived B cells (CD19^{+ve}, CD3^{-ve}), T cells (CD4^{+ve}, CD3^{+ve}), and Eosinophils (SiglecF^{+ve}, CD11b^{+ve}) showing *Cd45* surface expression in cells from *MRP8-Cre;Cd45*$^{fl/fl}$ mice compared to cells from *Cd45*$^{fl/fl}$ control mice. (C) Quantification of Flow cytometry data showing mean fluorescence intensity (n=3 independent experiments). Data represent means ± SEM and were analyzed by 1-way ANOVA followed by Tukey's post hoc testing.

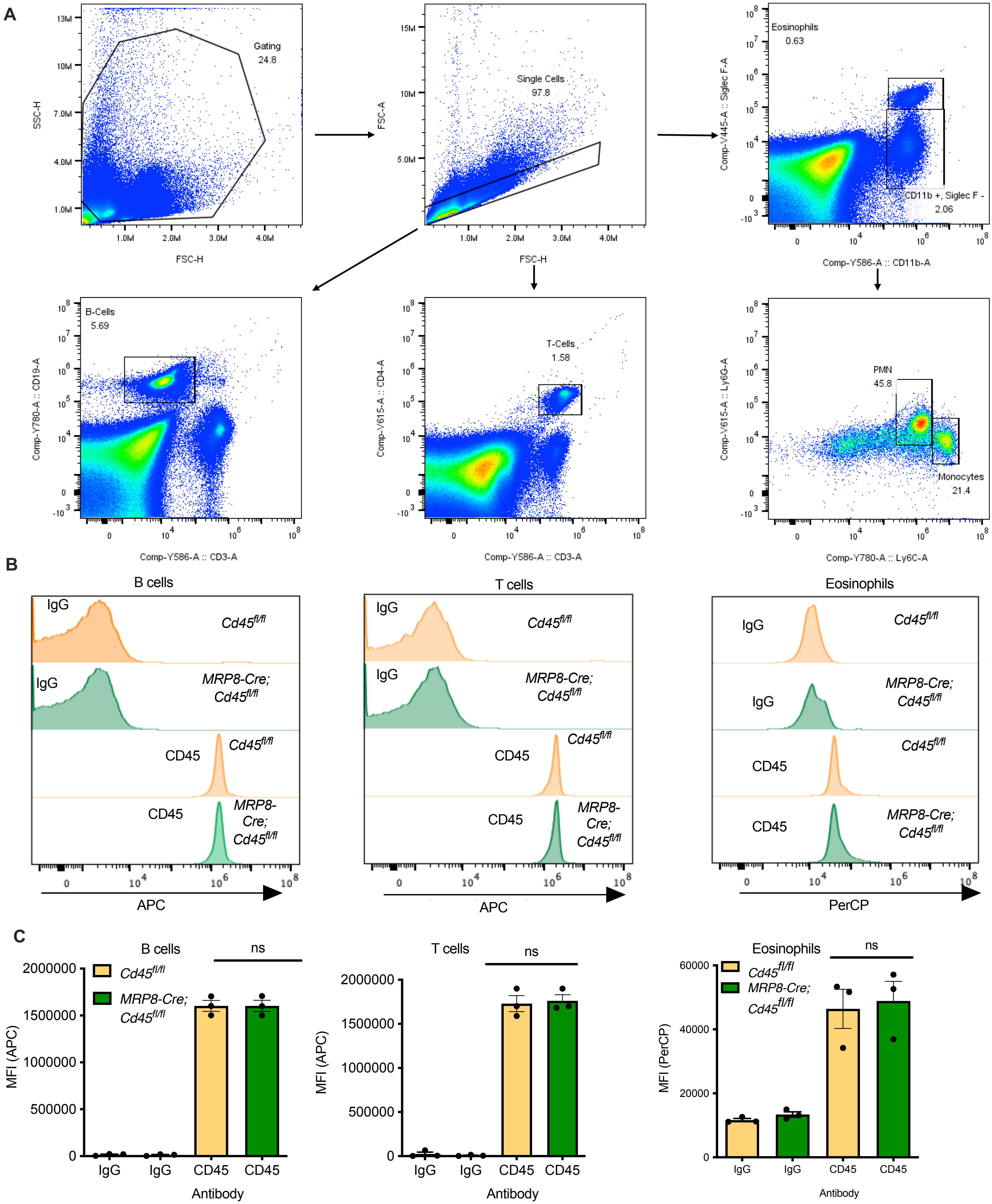

Supplemental Figure 7. CD45 Surface Expression on blood derived immune cells isolated from *Cd45*$^{fl/fl}$ mice and *MRP8-Cre;Cd45*$^{fl/fl}$ mice. (A) Gating strategy. (B) Representative flow cytometry plots of blood derived B cells (CD19^{+ve}, CD3^{-ve}), T cells (CD4$^{+ve,}$ CD3^{+ve}), and Eosinophils (SiglecF^{+ve}, CD11b^{+ve}) showing *Cd45* surface expression in cells from *MRP8-Cre;Cd45*$^{fl/fl}$ mice compared to cells from *Cd45*$^{fl/fl}$ control mice. (C) Quantification of Flow cytometry data showing mean fluorescence intensity (n =3 independent experiments). Data represent means ± SEM and were analyzed by 1-way ANOVA followed by Tukey's post hoc testing.

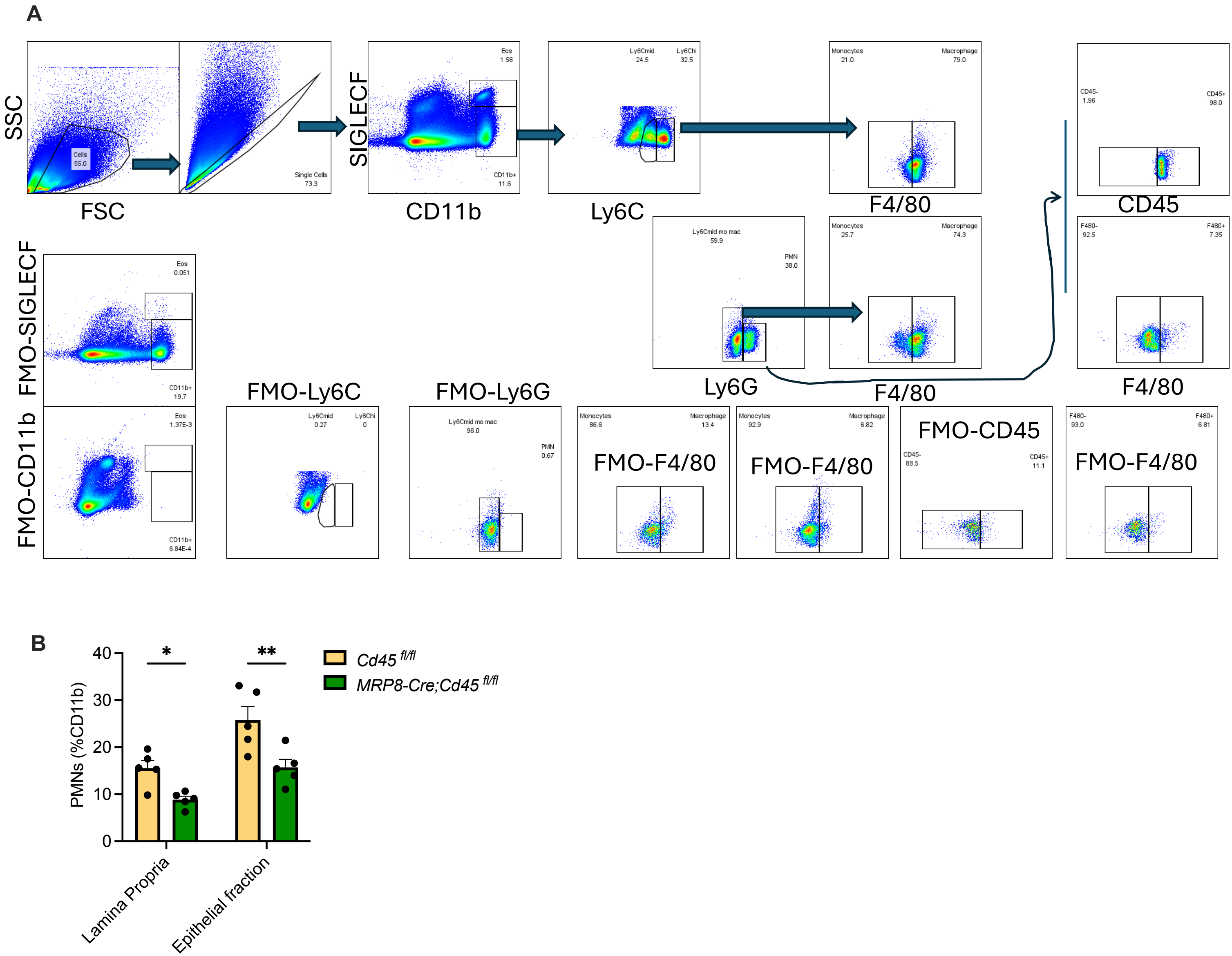

Supplemental Figure 8. Analysis of Lamina Propria Cells in *Cd45$^{fl/fl}$* mice and *MRP8-Cre;Cd45$^{fl/fl}$* mice. (A) Gating strategy for lamina propria cell identification by flow cytometry analysis. (B) Analysis of lamina propria infiltrating myeloid cells on day 8 revealed a significant decrease in colonic recruitment and epithelial association of neutrophils (Ly6G+) from *MRP8-Cre;Cd45$^{fl/fl}$* mice relative to *Cd45$^{fl/fl}$* mice. Numbers of cells were quantified by flow cytometry using counting beads. Values shown are percentages of PMN relative to total numbers of CD11b positive cells (C) (n=5 independent biological experiments, * $p<0.05$; ** $p<0.01$). Data represent means ± SEM and were analyzed by 1-way ANOVA followed by Tukey's post hoc testing.

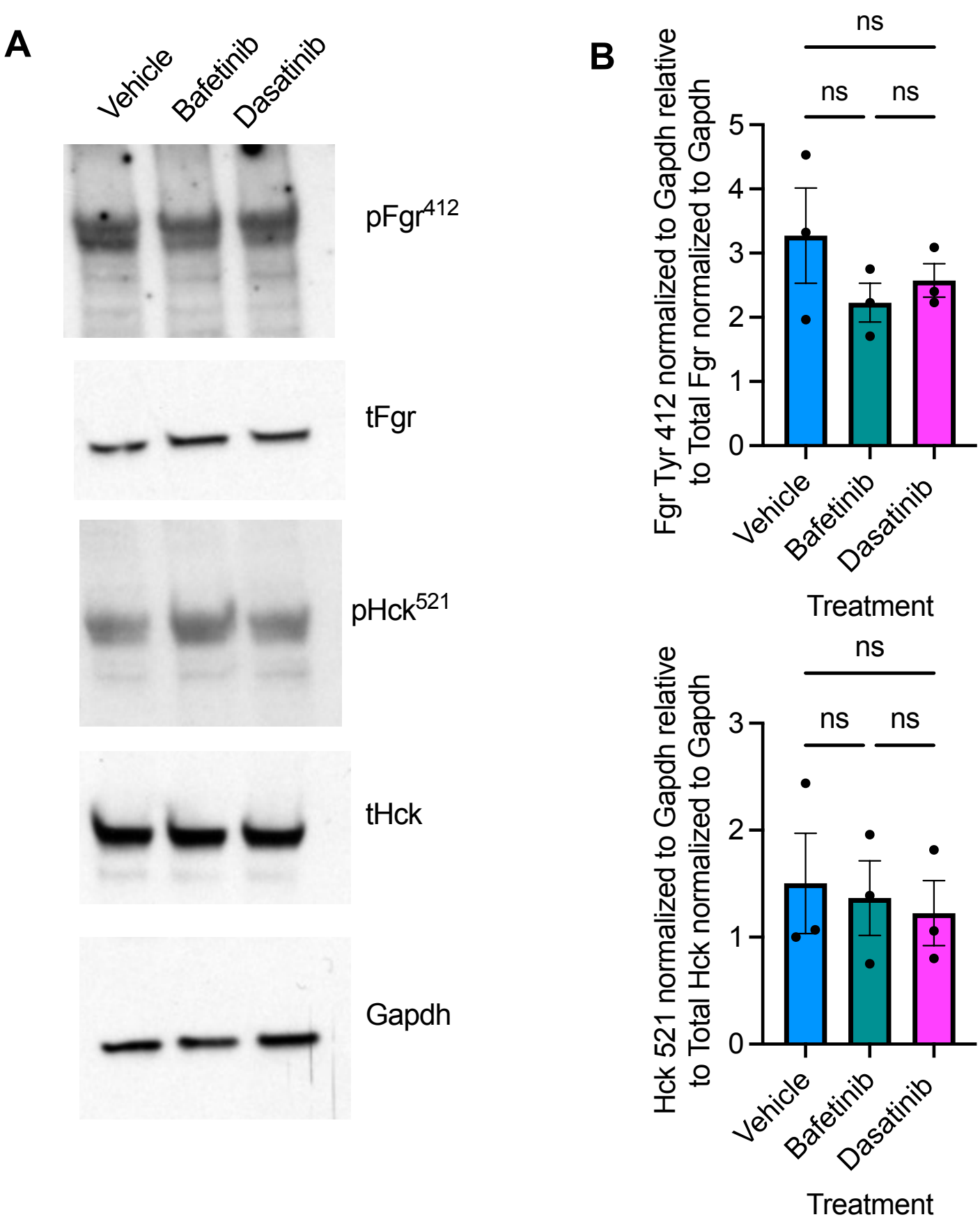

Supplemental Figure 9. Dasatinib and Bafetinib do no change phosphorylation of Hgr and Fck. Differentiated HL60 cells were stimulated with 100nM fMLF for 60 minutes and incubated with 250nM Dasatinib, 100nM Bafetinib or vehicle control. Representative blots are shown for indicated proteins or phosphoproteins. Blots are representative of n=3 independent experiments. (B) Densitometry showing no statistically significant changes in Hck or Fgr phosphorylation.
