# Supplementary material for: The protein tyrosine phosphatase CD45 promotes PMN transepithelial migration, antimicrobial function, and colonic mucosal repair
Source: JCI Insight. 2026 Jun 23;11(15):e203438. doi: 10.1172/jci.insight.203438 (PMC13463622; doi:10.1172/jci.insight.203438)
Supplement: Supplementary file 2 [file jciinsight-11-203438-s073.pdf]

Gapdh fig.4

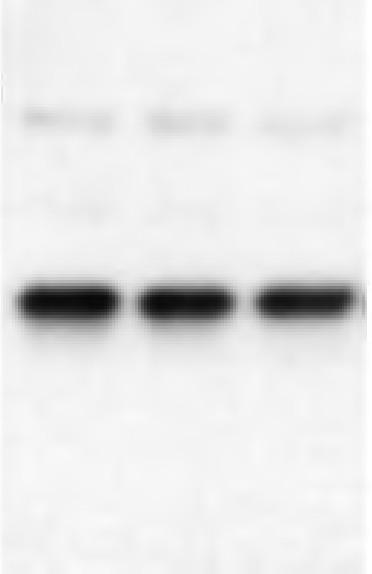

CD45

Fig.4

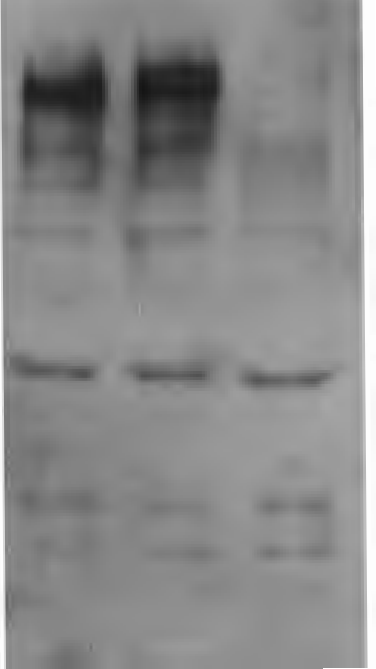

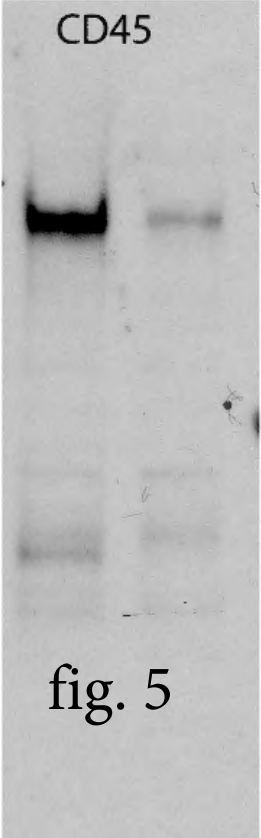

fig. 5

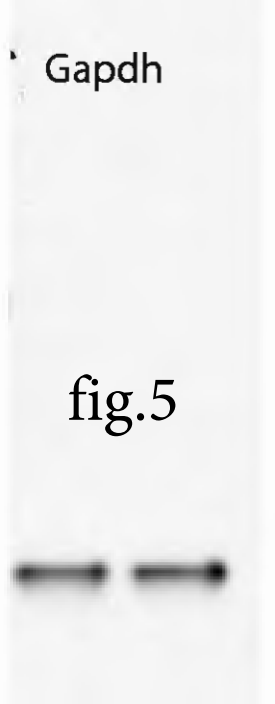

fig.5

Fig.10A

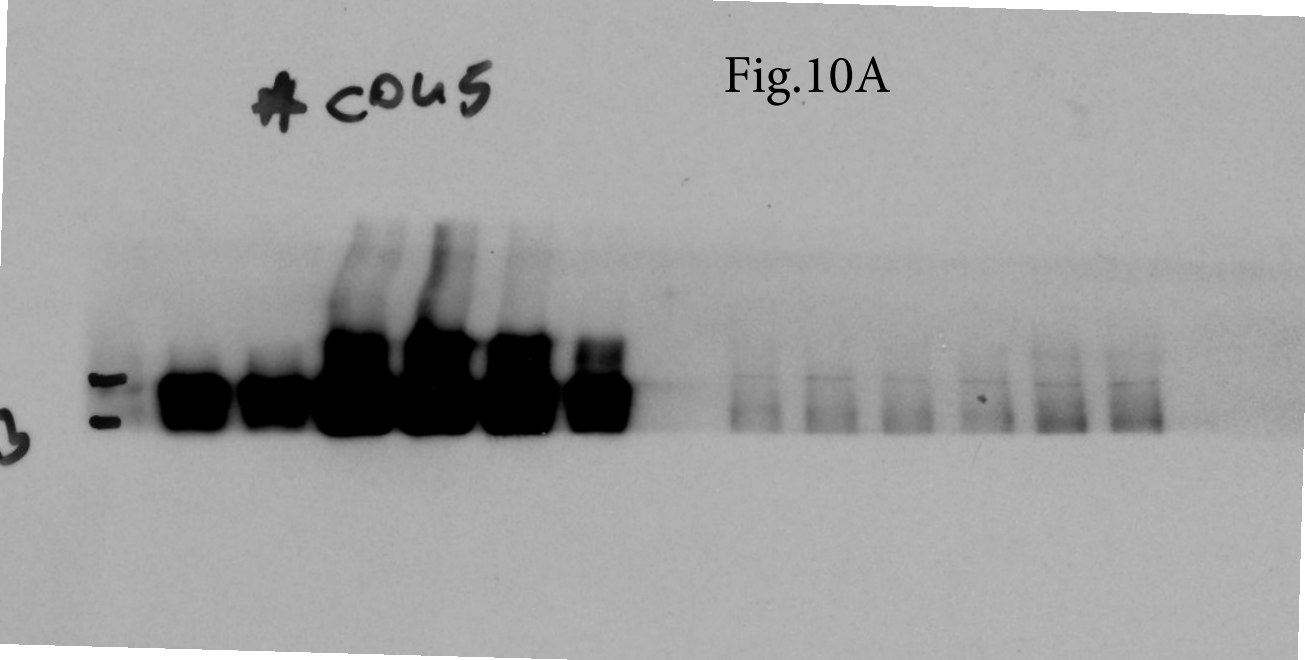

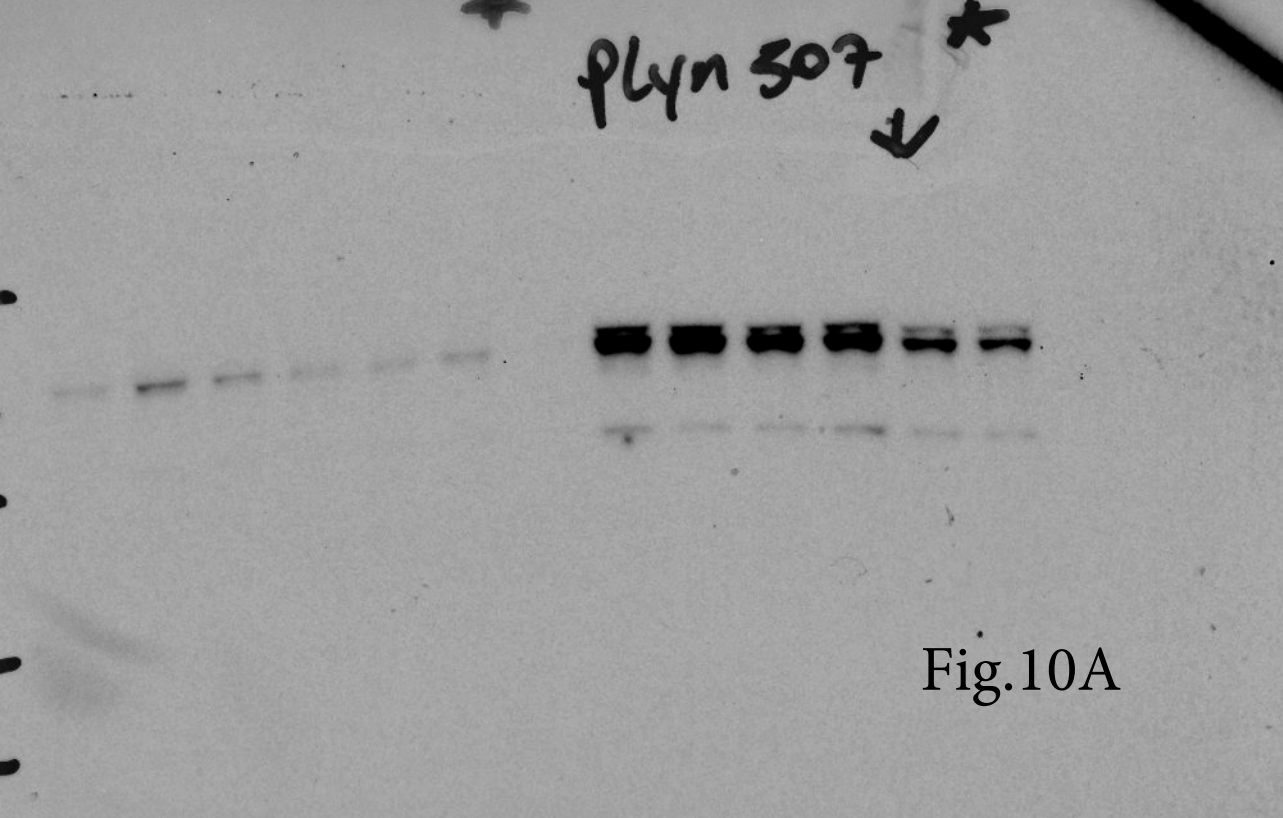

Fig.10A

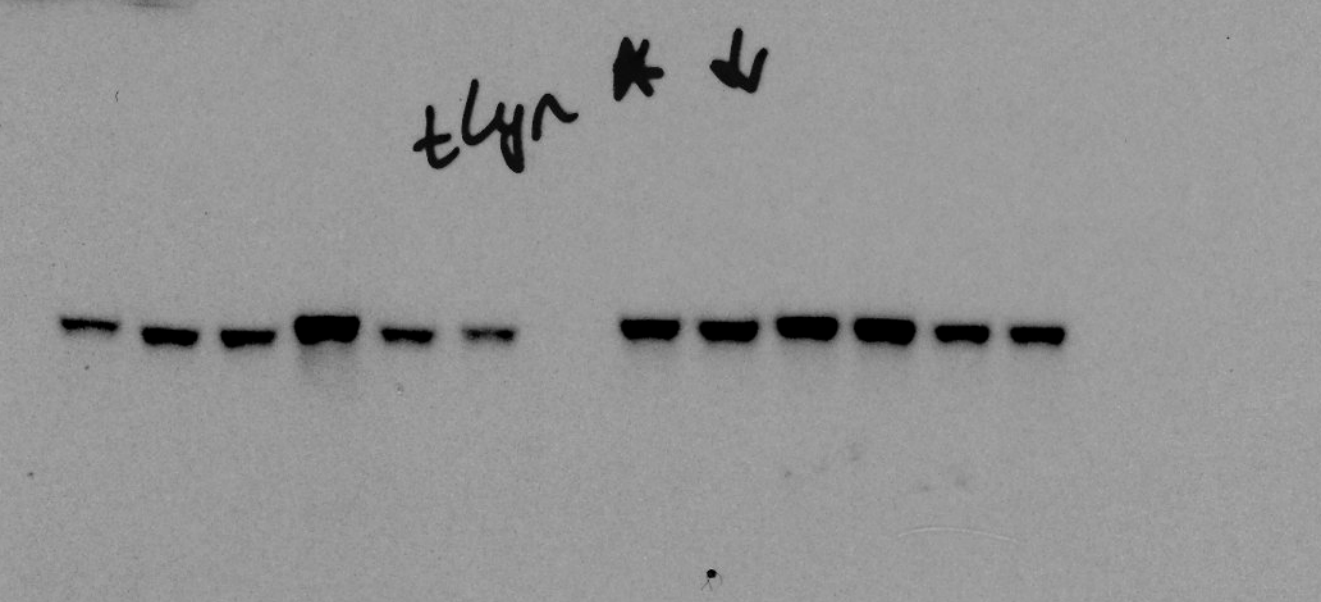

Fig.10A

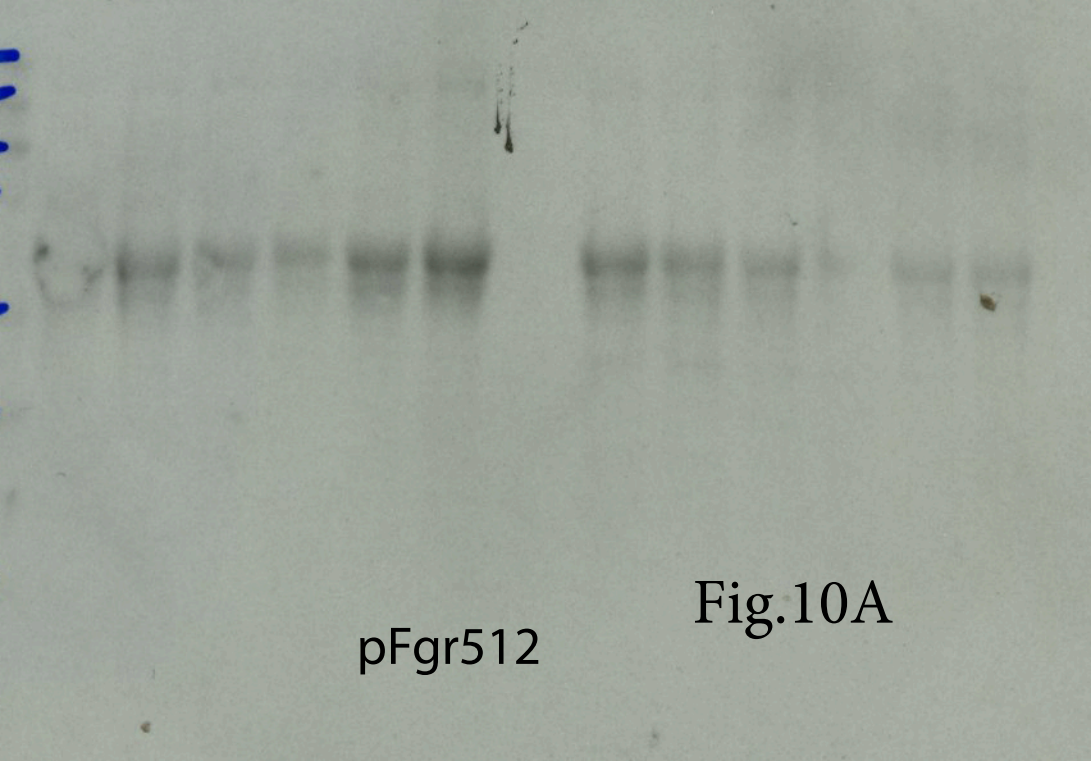

Fig.10A

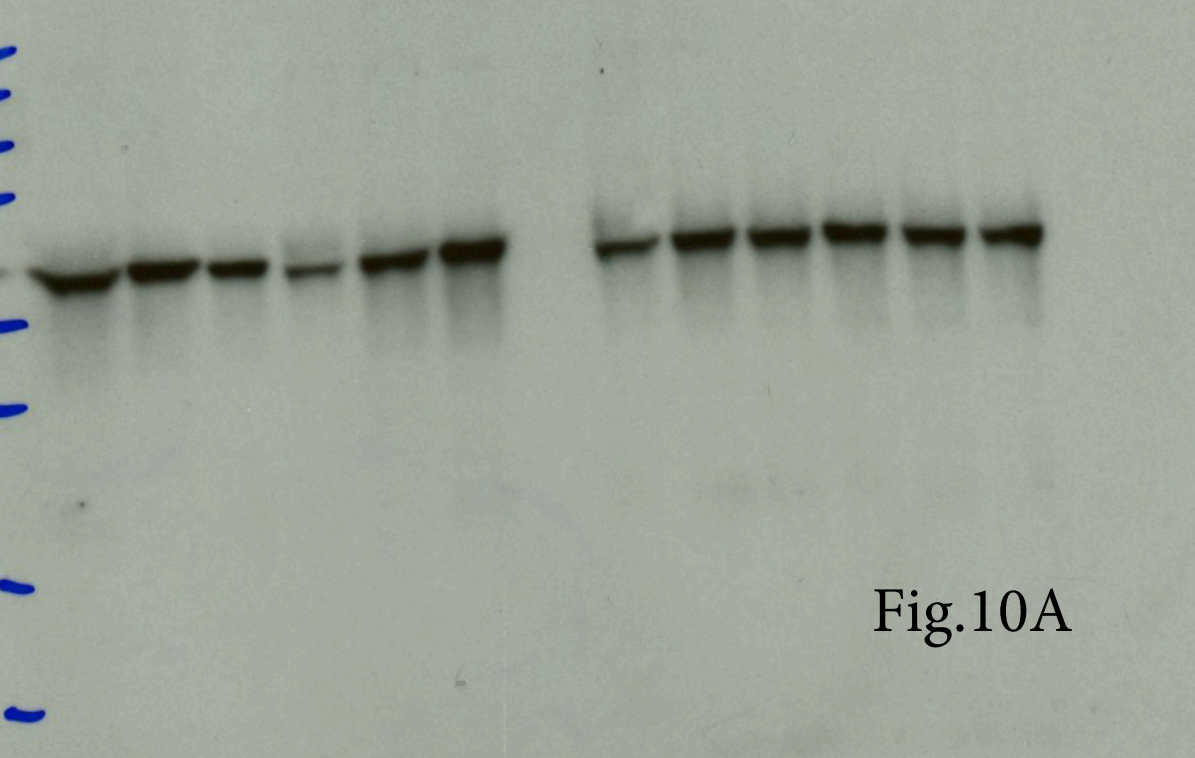

Fig.10A

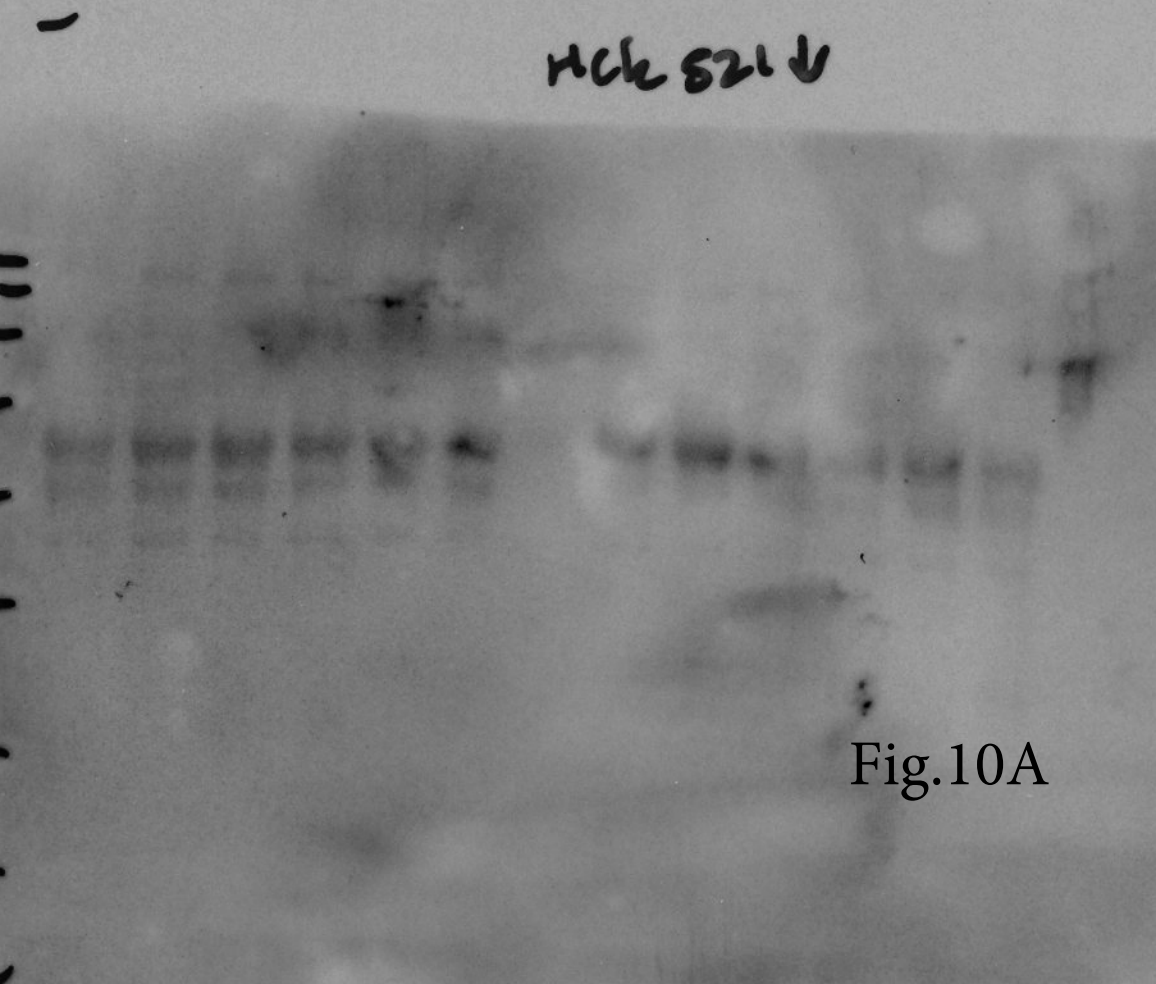

Fig.10A

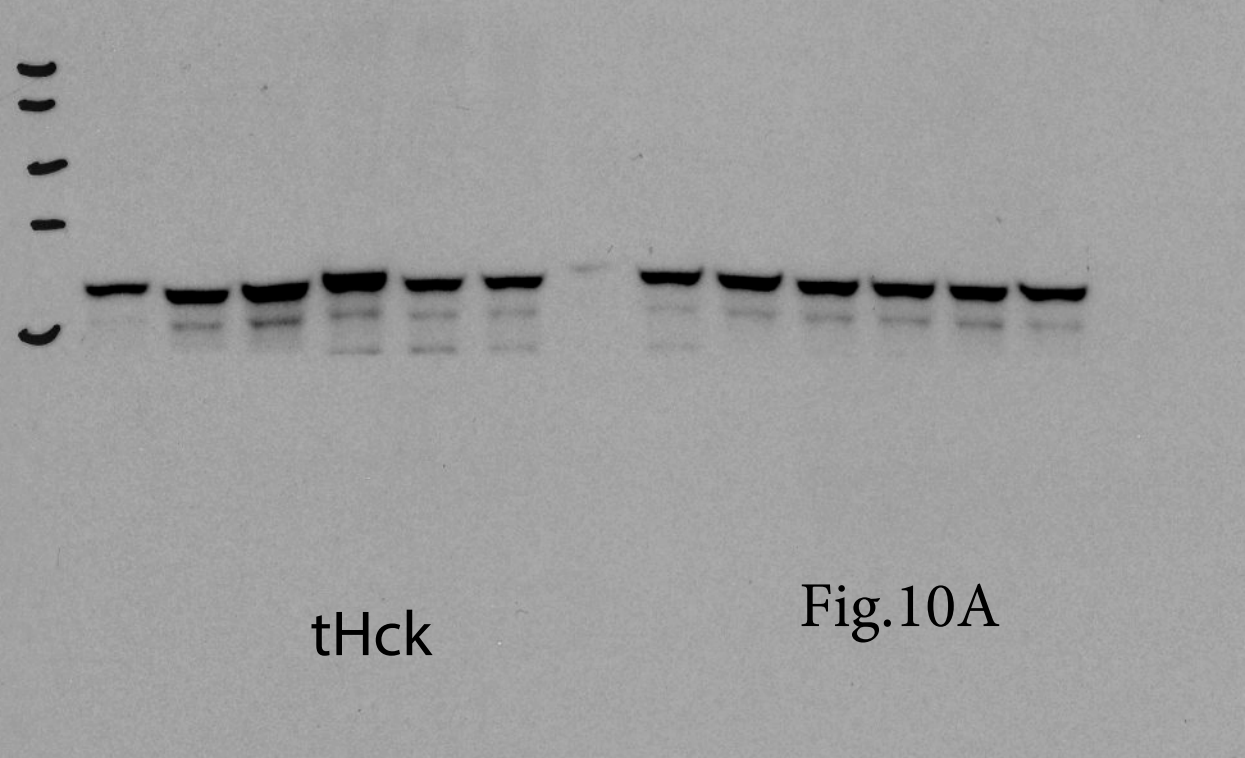

Fig.10A

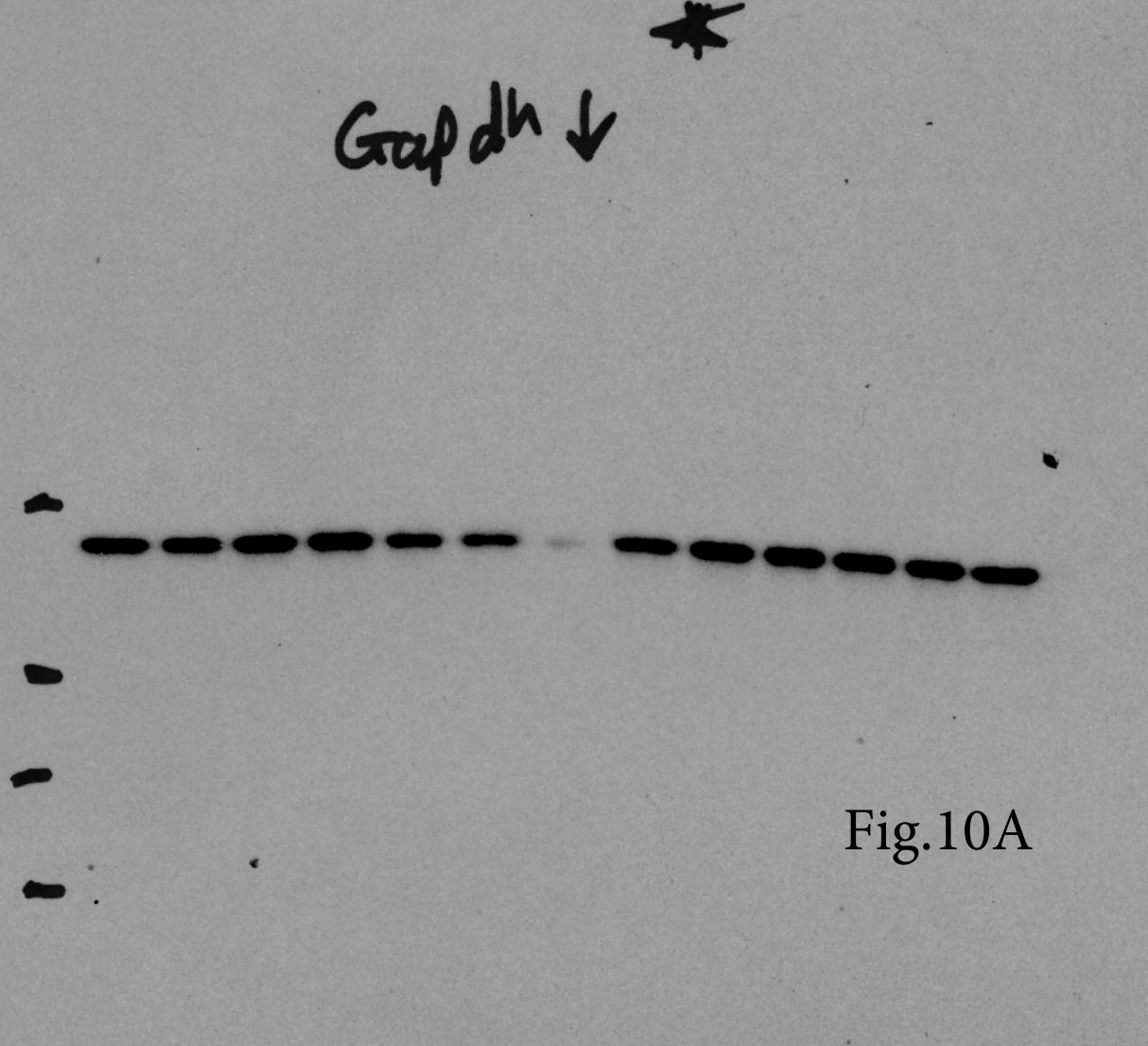

Fig.10A

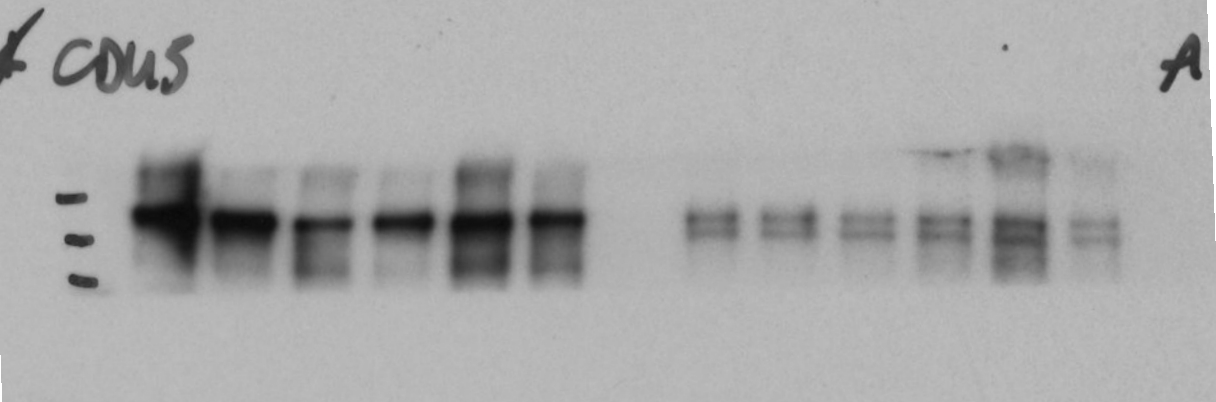

Fig.10C

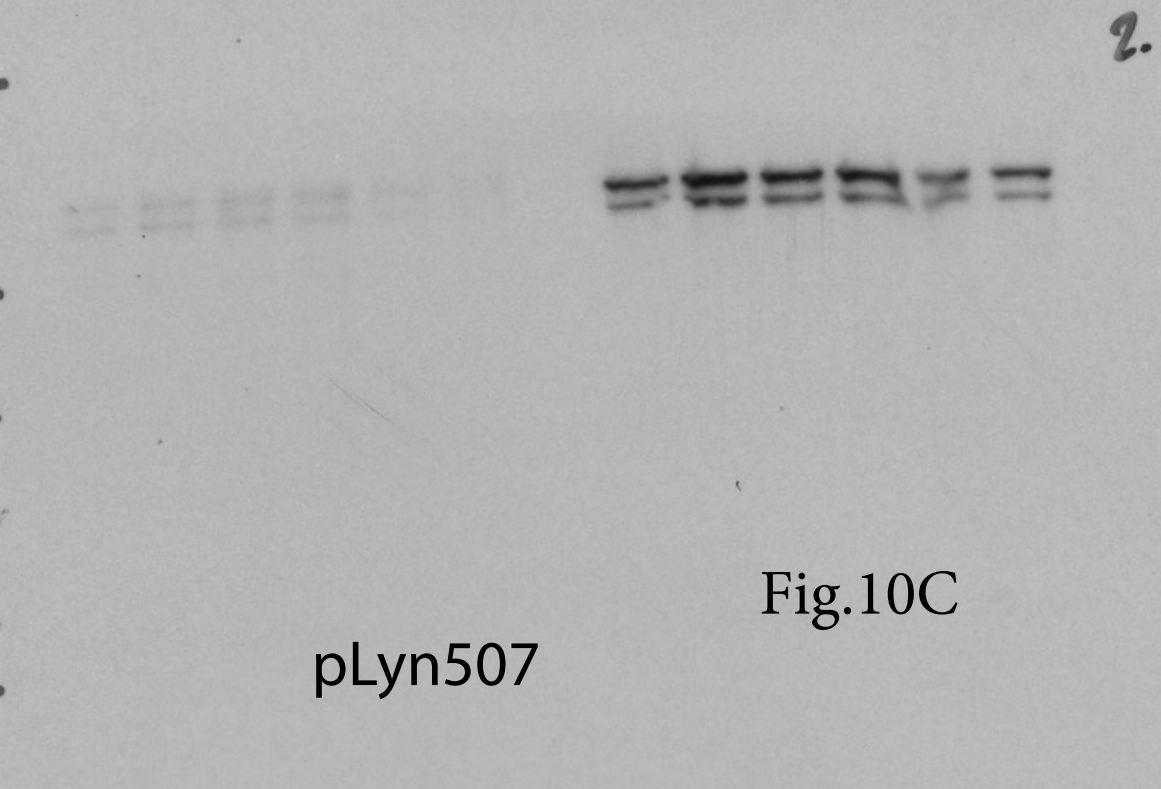

Fig.10C

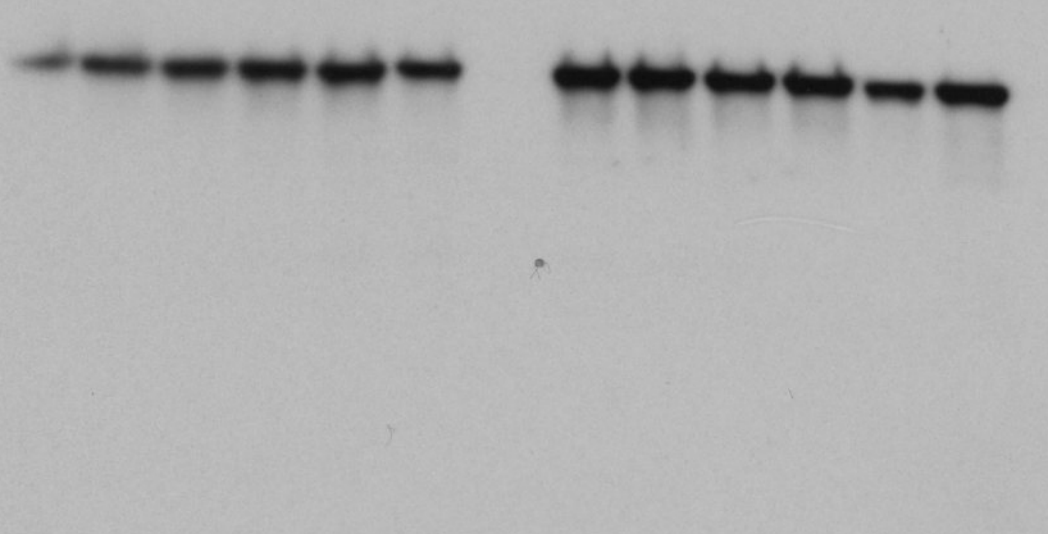

Fig.10C

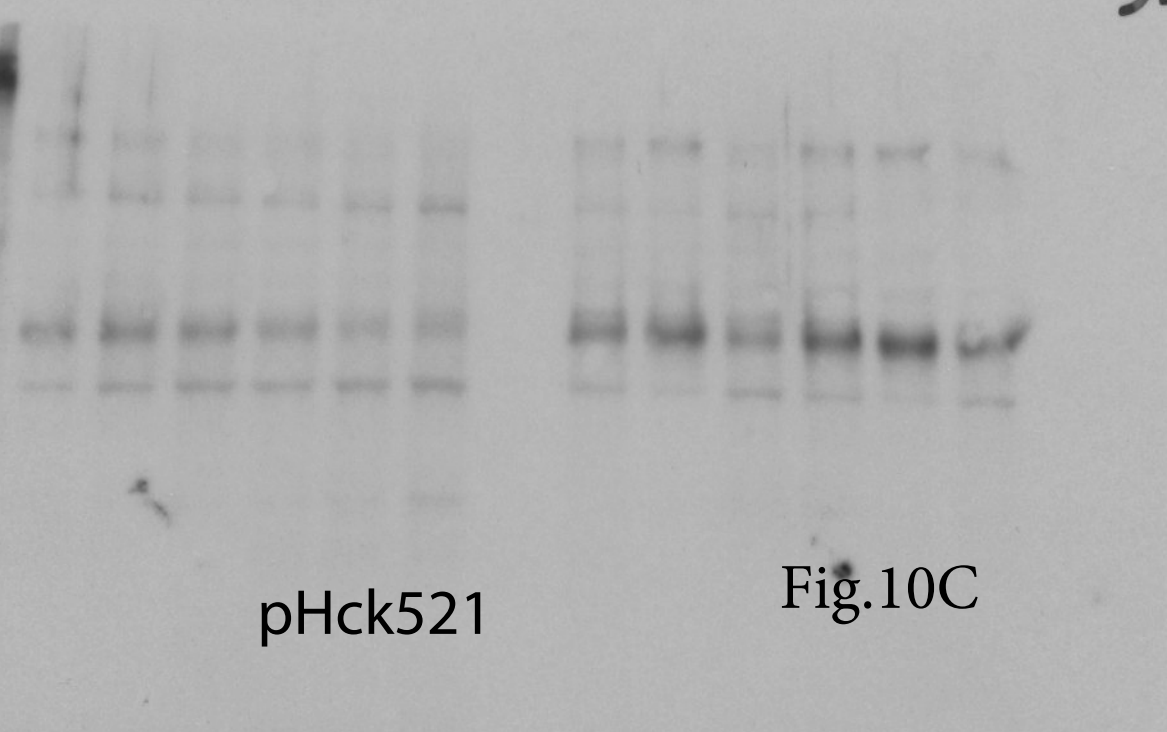

Fig.10C

Fig.10C

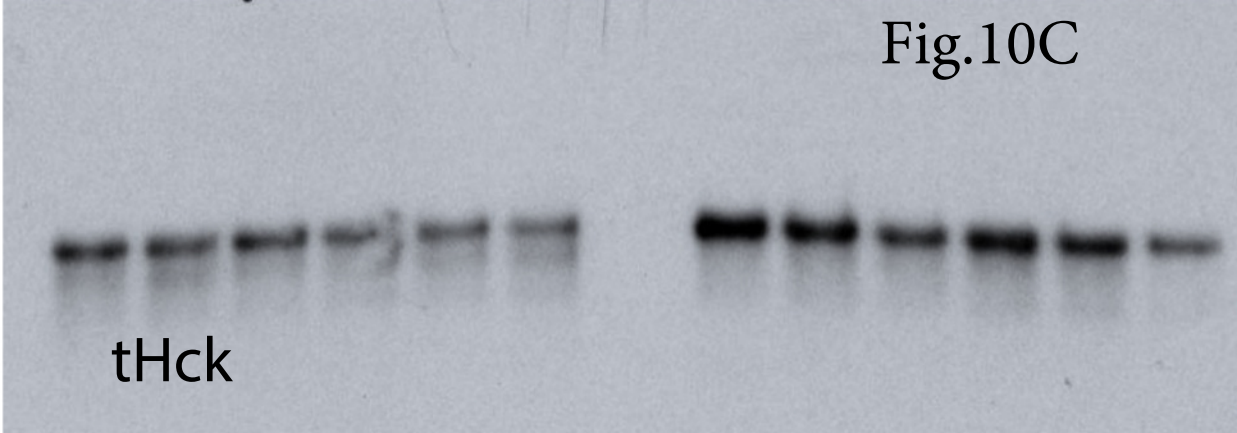

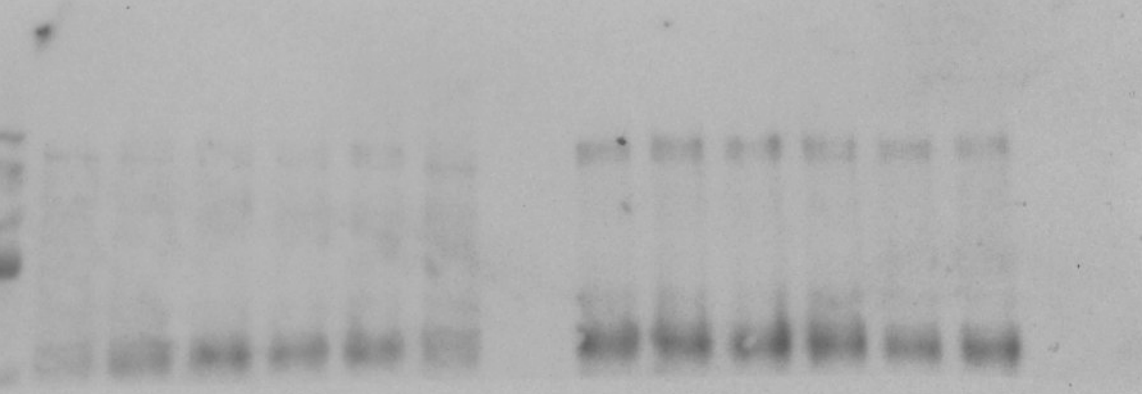

pFgr412

Fig.10C

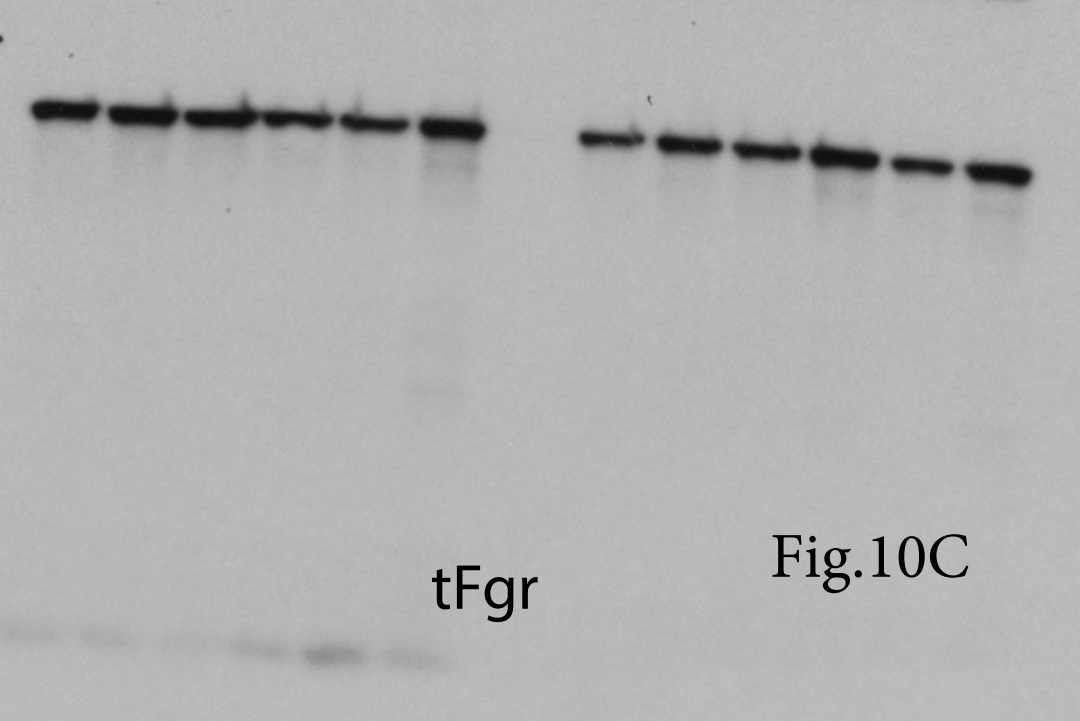

Fig.10C

Fig.10C

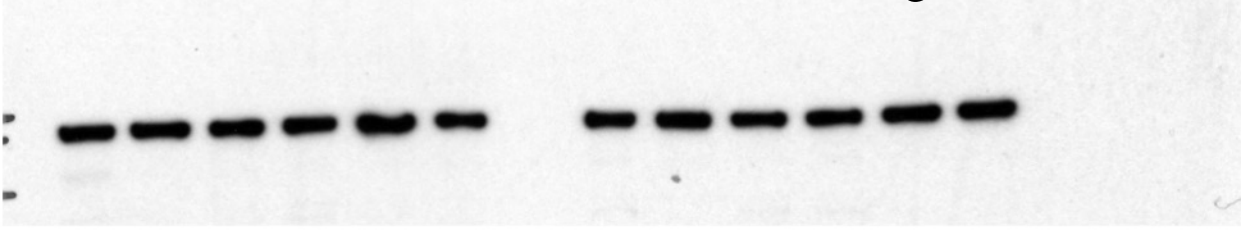

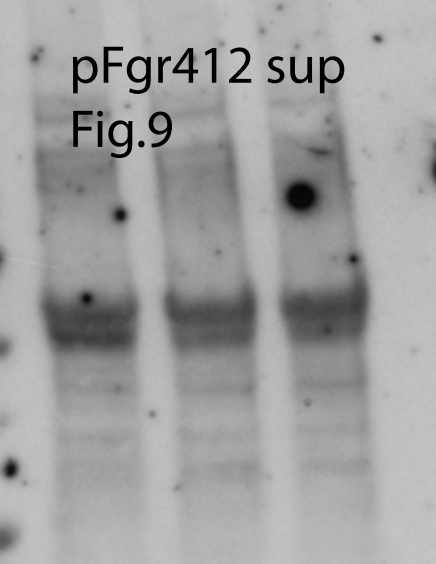
pFgr412 sup
Fig.9

tFgr Sup Fig.9

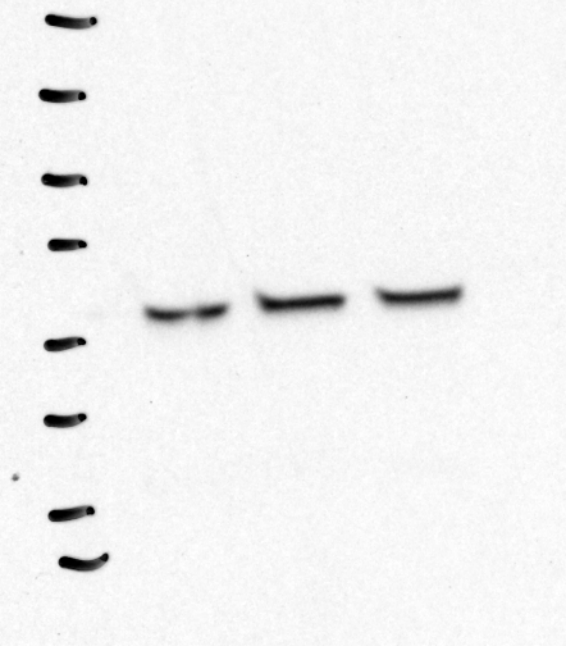

pHck521
Sup. Fig.9

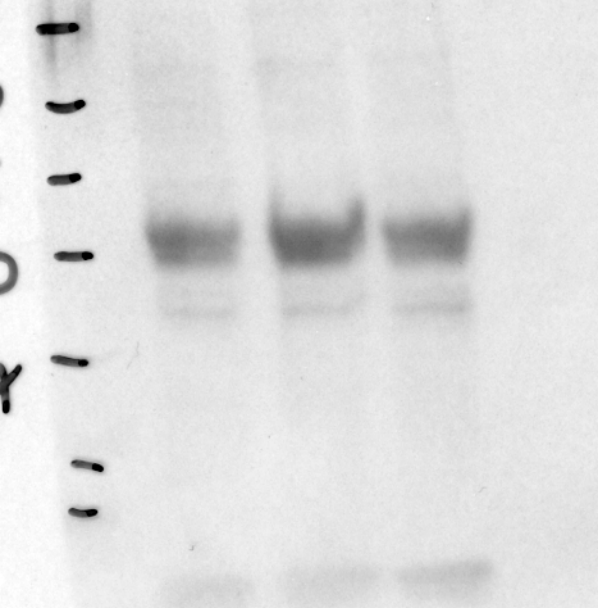

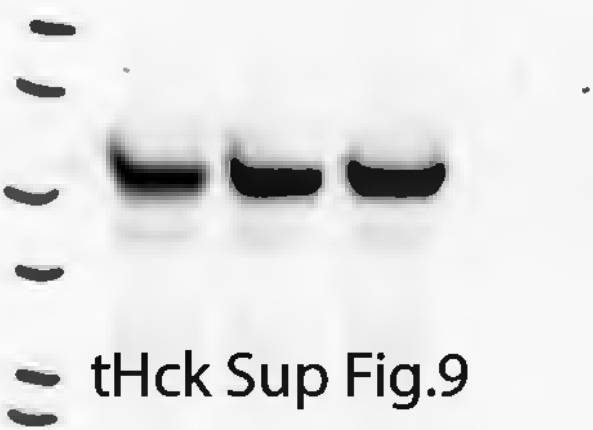

tHck Sup Fig.9

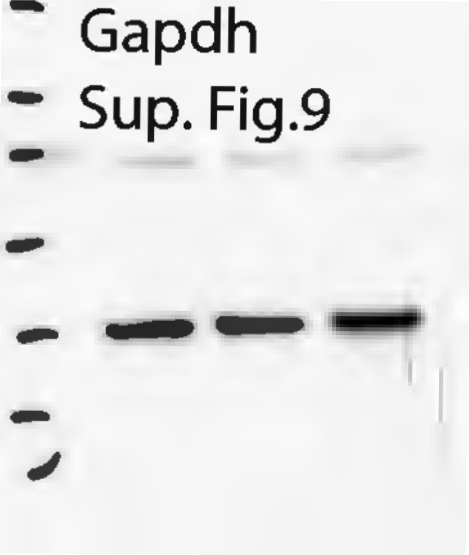
Gapdh
Sup. Fig.9
